# Supplementary material for: Comprehensive scoping review of health research using social media data
Source: BMJ Open. 2018 Dec 14;8(12):e022931. doi: 10.1136/bmjopen-2018-022931 (PMC6303712; doi:10.1136/bmjopen-2018-022931)
Supplement: Supplementary file 1 [file bmjopen-2018-022931supp001.pdf]

Appendix 1. Scoping and systematic reviews on the secondary use of data from social media

	Systematic or Quasi-Systematic Scoping Review	Review Aim/Objective/Main Focus	Number of Databases Used	Key Findings
1	Velasco, E., Agheneza, T., Denecke, K., Kirchner, G., Eckmanns, T.<br>Social media and internet-based data in global systems for public health surveillance: a systematic review. Milbank Q. 2014; 92(1): 7-33	To explore how useful are data from social media and the Internet, and what is the potential to enhance surveillance.	3	• No event-based surveillance systems currently used in national surveillance programs<br>• Existing systems have limitations<br>• There is a need for further development of automated technologies that monitor health-related information on the Internet<br>• The dissemination to health authorities of new information about health events is not always efficient and could be improved.<br>• No comprehensive evaluations show whether event-based surveillance systems have been integrated into actual epidemiological work during real-time health events.
2	Golder, S., Norman, G., Loke, Y.K.<br>Systematic review on the prevalence, frequency and comparative value of adverse events data in social media<br>British Journal of Clinical Pharmacology. 2015; 80(4); 878-88	To summarize the prevalence, frequency and comparative value of information on the adverse events of healthcare interventions from user comments and videos in social media.	16 databases and 2 internet search engines	• The prevalence of adverse events reports on social media varies from 0.2% to 8% of posts.<br>• 'Mild' and symptom-related adverse events are over-represented in social media and laboratory test abnormalities and 'serious' adverse events are under- represented compared with other data sources.<br>• The question as to whether searching social media for adverse events data is a valuable use of resources, resulting in improved patient outcomes remains unanswered.
3	Verhoef, L.M., Van de Belt, T.H.,<br>Engelen, L.J.L.P.G., Schoonhoven, L., Kool, R.B.<br>Social media and rating sites as tools to understanding quality of care: a scoping review<br>J Med Internet Res. 2014; 16(2); e56	To explore the relationship between data from social media and quality of care.	4	• There is a relationship between information on social media and quality of health care<br>• Drawbacks exist, since rating is anonymous and vulnerable to fraud. They are often based on only a few reviews and are predominantly positive.<br>• People providing feedback on health care via social media are presumably not always representative for the patient population.
4	Bernardo, T.M., Rajic, A., Young, I., Robiadek, K., Pham, M.T., Funk, J.A.<br>Scoping review on search queries and social media for disease surveillance: a chronology of innovation<br>J Med Internet Res. 2013; 15(7); e147	To assess the current state of knowledge regarding the use of search queries and social media for disease surveillance in order to inform future work on early detection and more effective mitigation of the effects of foodborne illness.	1 database and 2 internet search engines	• The use of search queries and social media for disease surveillance are relatively recent phenomena (first reported in 2006).<br>• Both the tools themselves and the methodologies for exploiting them are evolving over time.<br>• While their accuracy, speed, and cost compare favorably with existing surveillance systems, the primary challenge is to refine the data signal by reducing surrounding noise.
5	Fung, Tse, Cheung, Miu, & Fu. (2014). Ebola and the social media. The Lancet,	To systematically reviewed existing research pertinent to Ebola virus disease and social media, especially	6	• Included 7 articles on Twitter, 1 on Facebook, 3 on YouTube, 1 on Instagram & Flickr.

	Systematic or Quasi-Systematic Scoping Review	Review Aim/Objective/Main Focus	Number of Databases Used	Key Findings
	384(9961), 2207.	to identify the research questions and the methods used to collect and analyze social media.		• 11 articles examined Ebola posts' content themes, meta-data or user characteristics.<br>• One article examined how Ebola-related news videos influenced Twitter traffic.
6	Golder S, Ahmed S, Norman G, Booth A Attitudes Toward the Ethics of Research Using Social Media: A Systematic Review<br>J Med Internet Res 2017;19(6):e195	To ascertain attitudes on the ethical considerations of using social media as a data source for research as expressed by social media users and researchers.	16 data bases and 2 internet search engines	• Attitudes varied from overly positive with people expressing the views about the essential nature of such research for the public good, to very concerned with views that social media research should not happen.<br>• Underlying reasons for this variation related to issues such as the purpose and quality of the research, the researcher affiliation, and the potential harms.<br>• The methods used to conduct the research were also important.<br>• Many respondents were positive about social media research while adding caveats such as the need for informed consent or use restricted to public platforms only.
7	Kazemi, D., Borsari, B., Levine, M., & Dooley, B. (2017). Systematic review of surveillance by social media platforms for illicit drug use. Journal of Public Health (Oxford, England), 1-14.	To review the ability of social media to better recognize illicit drug use trends was addressed.	9	Selected studies used a range of social media tools/applications, including message boards, Twitter and blog/forums/platform discussions.
8	O'Shea, Jesse. (2017). Digital disease detection: A systematic review of event-based internet biosurveillance systems. International Journal of Medical Informatics, 101, 15-22.	To update the current state of knowledge on event-based Internet biosurveillance systems by identifying all systems, including current functionality, with hopes to aid decision makers with whether to incorporate new methods into comprehensive programmes of surveillance.	3	• Each system uses different innovative technology and data sources to gather data, process, and disseminate data to detect infectious disease outbreaks.
9	Yan, Chughtai, & Macintyre. (2017). Utility and potential of rapid epidemic intelligence from internet-based sources. International Journal of Infectious Diseases, 63, 77-87.	To identify and summarise the types of Internet-based surveillance methods studied in recent literature. A secondary aim was to identify and summarise the timeliness and accuracy outcomes of Internet-based methods described in literature.	5	• Studies used search queries, social media posts and approaches derived from existing Internet-based systems for early epidemic alerts and real-time monitoring.<br>• Most studies noted improved timeliness compared to official reporting<br>• Internet-based methods showed variable correlation strength with official datasets, with some methods showing reasonable accuracy.
10	Al-Garadi, Khan, Varathan, Mujtaba, & Al-Kabsi. (2016). Using online social networks to track a pandemic: A systematic review. Journal of Biomedical Informatics, 62, 1-11.	To conduct a systematic review of the use of online social networks to track a pandemic.	5	• Identified the extent of using online social networks to track a pandemic.<br>• Online social networks contain significant information to track a pandemic almost in real time.<br>• There are challenges and future implications.
11	Agheneza T. A Systematic Review of Event-Based Public Health Surveillance Systems. Hamburg: Faculty	To identify all the event-based surveillance systems that exist and where they are based.	3	• 13 event-based systems were identified, and with 10 of the 13 systems evaluated.<br>• N. America is the leading

	Systematic or Quasi-Systematic Scoping Review	Review Aim/Objective/Main Focus	Number of Databases Used	Key Findings
	of Life Sciences, Hamburg University of Applied Sciences; 2011.			continent with about 77 % of the event-based systems followed by Europe and lastly Asia. No system was found in Africa, Australia and S. America.
12	Emmert M, Sander U, Pisch F. Eight questions about physician-rating websites: a systematic review. J Med Internet Res. 2013;15(2):e24.	To identify frequently discussed issues about physician-rating websites.	7	• The current usage of physician-rating websites is still low but is increasing.<br>• International data show that 1 out of 6 physicians has been rated, and approximately 90% of all ratings on physician-rating websites were positive.<br>• Could not find any evidence of "doctor-bashing".<br>• Physicians should not ignore these websites, but rather, monitor the information available and use it for internal and external purposes.
13	Guy S, Ratzki-Leewing A, Bahati R, Gwadry-Sridhar F. Social media: A systematic review to understand the evidence and application in infodemiology. Lect Notes Inst Comput Sci Soc Informatics Telecommun Eng. 2012;91:1–8.	To systematically review the literature utilizing social media as a source for disease prediction and surveillance.	2	• Open-source micro-blogging sites can be used for influenza-like-illness monitoring.
14	Tursunbayeva, Franco, & Pagliari. (2017). Use of social media for e-Government in the public health sector: A systematic review of published studies. Government Information Quarterly, 34(2), 270-282.	To investigate the adoption and use of social media by public health organizations	4	• Evidence-base is mostly descriptive, unidisciplinary and lacks the theoretical depth seen in other branches of e-Government research.<br>• The lack of robust evidence makes it difficult to draw conclusions about the effectiveness of these approaches in the public health sector
15	Patel, Chang, Greysen, & Chopra. (2015). Social Media Use in Chronic Disease: A Systematic Review and Novel Taxonomy. The American Journal of Medicine, 128(12), 1335-1350.	To evaluate clinical outcomes from applications of contemporary social media in chronic disease; to develop a conceptual taxonomy to categorize, summarize, and then analyze the current evidence base; and to suggest a framework for future studies	1	• The overall impact of social media on chronic disease was variable.<br>• Among studies that showed benefit, most used either Facebook or blogs, and were based within the domain of support.
