# Supplementary material for: Comprehensive scoping review of health research using social media data
Source: BMJ Open. 2018 Dec 14;8(12):e022931. doi: 10.1136/bmjopen-2018-022931 (PMC6303712; doi:10.1136/bmjopen-2018-022931)
Supplement: Supplementary file 2 [file bmjopen-2018-022931supp002.pdf]

Appendix 2. Total number of publications by search query

**Search query**	**Total number of publications from 1997-2017**
"social media" AND (health OR illness OR disease) AND human	3425
digital AND surveillance AND (health OR illness OR disease) AND human	2935
facebook AND (health OR illness OR disease) AND human	939
twitter AND (health OR illness OR disease) AND human	709
"social media" AND surveillance AND (health OR illness OR disease) AND human	665
blog AND (health OR illness OR disease) AND human	602
"social media" AND epidemiology AND (disease OR health OR illness) AND human	581
"social network analysis" AND (disease OR health OR illness) AND human	462
youtube AND (health OR illness OR disease) AND human	351
"web 2.0" AND (health OR illness OR disease) AND human	312
facebook AND surveillance AND (health OR illness OR disease) AND human	206
twitter AND surveillance AND (health OR illness OR disease) AND human	187
digital AND ethnography AND (health OR illness OR disease) AND human	110
"online forum" AND (health OR illness OR disease) AND human	103
blog AND surveillance AND (health OR illness OR disease) AND human	76
patientslikeme AND (health OR illness OR disease) AND human	60
"online monitoring" AND (disease OR health OR illness) AND human	48
"health 2.0" AND (health OR illness OR disease) AND human	46
twitter AND mining AND (health OR illness OR disease) AND human	43
instagram AND (health OR illness OR disease) AND human	40
online AND "social network analysis" AND (disease OR health OR illness) AND human	29
"online forum" AND surveillance AND (health OR illness OR disease) AND human	25
"online community" AND surveillance AND (health OR illness OR disease) AND human	25
infoveillance AND (disease OR health OR illness) AND human	24
"online surveillance" AND (disease OR health OR illness) AND human	23
"social network analysis" AND web AND (disease OR health OR illness) AND human	23
"digital surveillance" AND (disease OR health OR illness) AND human	16
netnography AND (disease OR health OR illness) AND human	14
"medicine 2.0" AND (health OR illness OR disease) AND human	14
"social media analysis" AND (disease OR health OR illness) AND human	12
"online tracking" AND (disease OR health OR illness) AND human	12
"web analytics" AND (disease OR health OR illness) AND human	10
"social media analytics" AND (disease OR health OR illness) AND human	9
"social media monitoring" AND (disease OR health OR illness) AND human	9
blog AND mining AND (health OR illness OR disease) AND human	9
"web mining" AND (disease OR health OR illness) AND human	8
"digital monitoring" AND (disease OR health OR illness) AND human	7
facebook AND mining AND (health OR illness OR disease) AND human	7
"social media mining" AND (disease OR health OR illness) AND human	6
"web monitoring" AND (disease OR health OR illness) AND human	6

**Search query**	**Total number of publications from 1997-2017**
"social media platform" AND surveillance AND (health OR illness OR disease) AND human	6
"social media surveillance" AND (disease OR health OR illness) AND human	4
"web tracking" AND (disease OR health OR illness) AND human	4
digital AND "social network analysis" AND (disease OR health OR illness) AND human	4
"social network analysis" AND digital AND (disease OR health OR illness) AND human	4
"digital ethnography" AND (health OR illness OR disease) AND human	4
"digital tracking" AND (disease OR health OR illness) AND human	3
"online analytics" AND (disease OR health OR illness) AND human	3
"social media site" AND surveillance AND (health OR illness OR disease) AND human	3
"social media listening" AND (disease OR health OR illness) AND human	2
"social network analytics" AND (disease OR health OR illness) AND human	2
"social media website" AND surveillance AND (health OR illness OR disease) AND human	2
"web surveillance" AND (disease OR health OR illness) AND human	0
"social network surveillance" AND (disease OR health OR illness) AND human	0
"social media observing" AND (disease OR health OR illness) AND human	0
"social media tracking" AND (disease OR health OR illness) AND human	0
"digital observing" AND (disease OR health OR illness) AND human	0
"digital analytics" AND (disease OR health OR illness) AND human	0
"digital mining" AND (disease OR health OR illness) AND human	0
"digital listening" AND (disease OR health OR illness) AND human	0
"web observing" AND (disease OR health OR illness) AND human	0
"web listening" AND (disease OR health OR illness) AND human	0
"online observing" AND (disease OR health OR illness) AND human	0
"online mining" AND (disease OR health OR illness) AND human	0
"online listening" AND (disease OR health OR illness) AND human	0
"social network observing" AND (disease OR health OR illness) AND human	0
"social network mining" AND (disease OR health OR illness) AND human	0
"social network monitoring" AND (disease OR health OR illness) AND human	0
"social network listening" AND (disease OR health OR illness) AND human	0
"social network tracking" AND (disease OR health OR illness) AND human	0
linkedin AND (health OR illness OR disease) AND human	0
"sixdegrees" AND (health OR illness OR disease) AND human	0
