# Supplementary material for: Comprehensive scoping review of health research using social media data
Source: BMJ Open. 2018 Dec 14;8(12):e022931. doi: 10.1136/bmjopen-2018-022931 (PMC6303712; doi:10.1136/bmjopen-2018-022931)
Supplement: Author's manuscript [file bmjopen-2018-022931.draft_revisions.pdf]

BMJ Open is committed to open peer review. As part of this commitment we make the peer review history of every article we publish publicly available.

When an article is published we post the peer reviewers' comments and the authors' responses online. We also post the versions of the paper that were used during peer review. These are the versions that the peer review comments apply to.

The versions of the paper that follow are the versions that were submitted during the peer review process. They are not the versions of record or the final published versions. They should not be cited or distributed as the published version of this manuscript.

BMJ Open is an open access journal and the full, final, typeset and author-corrected version of record of the manuscript is available on our site with no access controls, subscription charges or pay-per-view fees (http://bmjopen.bmj.com).

If you have any questions on BMJ Open's open peer review process please email info.bmjopen@bmj.com

BMJ Open

A Comprehensive Scoping Review of Health Research Using Social Media Data (Protocol)

Journal:	*BMJ Open*
Manuscript ID	bmjopen-2018-022931
Article Type:	Protocol
Date Submitted by the Author:	16-Mar-2018

Complete List of Authors:	Taylor, Joanna; University of Edinburgh, eHealth Research Group, Usher Institute of Population Health Sciences and Informatics; Ernst and Young<br><br>Pagliari, Claudia; University of Edinburgh, eHealth Research Group, Usher Institute for Population Health Sciences

Keywords:	Social Media, eHealth, Digital Research, Public Health Informatics, Data Science

SCHOLARONE™
Manuscripts

A Comprehensive Scoping Review of Health Research Using Social Media Data (Protocol)

Joanna Taylor and Claudia Pagliari

Corresponding Author: Claudia Pagliari, eHealth Research Group, Usher Institute for Population Health Sciences and Informatics, University of Edinburgh, Medical School, Teviot Place, Edinburgh EH8 9AG, UK.
Email: claudia.pagliari@ed.ac.uk
Tel: 0131 6509464
Fax: 0131 6509119

Joanna Taylor
Usher Institute for Population Health Sciences and Informatics, The University of Edinburgh, UK
Ernst and Young Ltd, Switzerland

Claudia Pagliari
Usher Institute for Population Health Sciences and Informatics, The University of Edinburgh, UK
claudia.pagliari@ed.ac.uk

Keywords: Social Media [MeSH]**, eHealth, Digital Research, Public Health Informatics** [MeSH]**, Data Science**

Word count: 2823 (excluding tables)

For peer review only

Abstract

Introduction: The rising popularity of social media, since their inception around 20 years ago, is reflected in the growth of health-related research using data derived from them. This has created a demand for literature reviews to synthesise this emerging evidence-base and inform future activities. Existing reviews tend to be narrow in scope, with limited consideration of the different types of data, analytical methods and ethical issues involved. There has also been a tendency for research to be siloed within different academic communities (e.g. computer science, public health), hindering knowledge translation. To address these limitations, we will undertake a comprehensive scoping review, to systematically capture the broad corpus of published, health-related research based on social media data. Here we present the review protocol and the pilot analyses used to inform it.

Methods: A five-stage scoping review framework will be followed: (1) identifying the research question; (2) identifying the relevant literature; (3) selecting the studies; (4) charting the data; and (5) collating, summarizing and reporting the results. To inform the literature search strategy we developed and tested an inclusive set of keyword combinations related to social media, health and relevant methodologies. This revealed the changing usage of relevant terms over time, the types of keyword combinations most likely to yield eligible studies, and the time-lag between the launch of new social media platforms and published research using data from them. In the scoping review three leading health, informatics, business and cross-disciplinary databases will be searched: PubMed, Scopus and Applied Social Sciences Index and Abstracts, alongside the Google search engine. There will be no restriction by date.

Ethics and dissemination: This review focuses on published research and no ethics approval is required. It will be submitted for publication to a peer-reviewed, interdisciplinary Open Access journal, and conferences on public health and digital research.

Article Summary

Strengths and limitations of this study

- The proposed scoping review addresses the need for a comprehensive, cross-disciplinary synthesis of health-related research using social media data.
- A 5-stage scoping review protocol was developed, which was informed by an exploratory analysis of existing reviews, terminologies and published taxonomies.
- This revealed the changing usage of relevant terms over time, the types of keyword combinations most likely to yield eligible studies, and the time-lag between the launch of new social media platforms and published research using data derived from them.
- The findings of the scoping review will be communicated using both static and multi-media visualisation tools.

For peer review only

Introduction - Context and coverage of the existing review literature

Social media are online, often mobile, platforms that support the creation and exchange of user-generated content [1]. They include generic platforms for networking, information sharing and collaboration (e.g. Facebook, Twitter, YouTube, LinkedIn) and online forums aimed at specific communities (e.g. PatientsLikeMe, Mumsnet). The first modern day social media platform, Sixdegrees, was launched in 1997 and allowed users to connect with friends and family through sending messages and posts on bulletin boards [2]. Since then the use of social media has become increasingly common [3] with an estimated 2.5 billion of the global population estimated to be using them as of 2017 [4].

As social media have become an integral part of people's lives, research focusing on social media has also evolved and is taking place in a number of domains. A bibliometric content analysis of key words that appeared in the abstracts of 14,500 journal articles and conference papers identified five main areas in which a high volume of research already exists. This includes research related to health, educational uses of social media, computing and computer science methods, business, organizational and marketing topics, and political and social engagement [5].

Uses of social media in the context of research can be described according to the broad categories identified by Bjerglund-Andersen and Söderqvist (dissemination, discussion/networking, public engagement, teaching, research/data collection) [6]. Taylor and Pagliari (2017) [7] recently separated the last of these into social media as a *source of data* for research and as a tool for *the conduct of research*. These 'secondary uses' of social media data include analyses of trends, associations and sentiments in users' postings, as well as interactions and networks [8, 9].

This new source of 'big data' has triggered scientific developments in a number of areas, including health. The term "infoveillance" was coined by Eysenbach in 2009 to describe the automated harvesting and analysis of internet searches and social media postings as an alternative approach for health and disease surveillance [10]. Other terms have since been introduced across academia and industry to describe the use of such data for gleaning insights about behavioural trends, determining the impact of interventions or predicting future events. These include "social media listening" [11], "social media mining" [12], "social analytics" [13], "social machines" [14] and "netnography" [15], to name but a few.

Based on an iterative search using PubMed we identified an existing corpus of 15 systematic, quasi-systematic and scoping reviews on the secondary use of social media data for health research, examples of which are summarized in Table 1 [see Appendix 1 for the full list, summarised according to their aims, number of databases and findings]. Amongst these are reviews on approaches to health surveillance [16-23] quality of healthcare services [24, 25], adverse event monitoring [26], illicit drug use [27], eGovernment [28], chronic conditions [29] and ethics [30].

Table 1. Existing systematic, quasi-systematic or scoping reviews indexed in PubMed.

Topic	Examples of Systematic, Quasi-systematic or Scoping Reviews
Disease surveillance	Social media and internet-based data in global systems for public health surveillance: a systematic review [16]
	Scoping review on search queries and social media for disease surveillance: a chronology of innovation [17]
	Ebola and the social media. [18]
	Digital disease detection: A systematic review of event-based internet biosurveillance systems. [19]
	Utility and potential of rapid epidemic intelligence from internet-based sources. [20]
	Using online social networks to track a pandemic: A systematic review. [21]
	A Systematic Review of Event-Based Public Health Surveillance Systems. [22]
	Social media: A systematic review to understand the evidence and application in infodemiology. [23]
Adverse event monitoring	Systematic review on the prevalence, frequency and comparative value of adverse events data in social media [26]
Quality of healthcare services	Social media and rating sites as tools to understanding quality of care: a scoping review [24]
	Eight questions about physician-rating websites: a systematic review. [25]
Illicit drug use	Systematic review of surveillance by social media platforms for illicit drug use. [27]
eGovernment	Use of social media for e-Government in the public health sector: A systematic review of published studies. [28]
Chronic disease	Social Media Use in Chronic Disease: A Systematic Review and Novel Taxonomy [29]
Ethics	Attitudes Toward the Ethics of Research Using Social Media: A Systematic Review [30]

A 2013 scoping review by Bernardo et al. on the use of internet search queries and social media for disease surveillance identified the first study as has having been published in 2006 and described how techniques for exploiting this data are evolving to increase the accuracy of signal detection [17]. Although insightful in understanding the chronology of this type of innovation, the review searched only 1 database and focused on the surveillance of foodborne illness. A 2014 systematic review by Velasco et al. on the impact and use of data from social media for public health surveillance went further, concluding that existing surveillance systems are limited and there is a need for automated technologies to monitor health related information on the Internet, although it did not specify which types of systems were analysed [16]. O'Shea et al.'s 2017 systematic review of event-based internet biosurveillance systems describes the wide variety of technologies and data sources for gathering, processing and disseminating data to detect infectious disease outbreaks [19]. These reviews, alongside the others listed, focus predominantly on infectious disease. A systematic review on social media for chronic disease exists [29] which focuses on understanding the clinical outcomes associated with using such technologies for patient support, education and disease management across different conditions. However this is limited by the inclusion of only database, and did not examine the type of methods or tools used to extract and analyse social media data, the academic discipline and setting of the research or the ethical issues considered. With respect to the latter, a recent systematic review by Golder et al., analysed studies reporting attitudes to research using social media data. This revealed wide variation in attitudes, from the very positive to the very concerned, depending on the purpose and quality of the research, researcher affiliation, the potential for harm and the methods used [30]. Although it used an impressive 16 databases, this review did not examine regional, disciplinary, condition or health topic specific variations and the authors note that the demographic characteristics of respondents was unclear in most studies. As noted in a recent review of UK research ethics guidelines and published health research, growing public awareness of the misuse of social media for marketing and algorithmic prediction, are forcing policymakers to look more closely at this issue [7].

In light of these gaps, we set out to undertake a comprehensive scoping review aimed at capturing and profiling a broad corpus of published multi-disciplinary research in which data obtained from social media have been used to monitor, understand or evaluate aspects of health and disease. In this paper, we describe the formal protocol for the comprehensive scoping review, alongside the preliminary analyses undertaken to inform each stage.

Methods and analysis

Scoping reviews are a type of quasi-systematic review that are increasingly used for understanding research on emerging innovations, which may be poorly indexed, distributed across published and grey literature or located in different academic disciplines [31-33]. They typically progress in five key stages (1) identifying the research question; (2) identifying the relevant studies; (3) selecting the studies; (4) charting the data; and (5) collating, summarizing and reporting the results. Developing an *a priori* review protocol can be useful for managing this complexity, while formative research can aid the design of such protocols by identifying relevant terminologies, topics and evidence sources. Scoping reviews are mainly aimed at mapping the evidence landscape rather than establishing the effectiveness of particular interventions and typically do not involve critical appraisal of study methodology or detailed extraction of outcomes data [34].

Stage 1: identifying the research question

In addition to the overarching review objective articulated in the introduction, several specific questions will be used to guide our analysis of existing research evidence, as listed in Table 2.

Table 2. Research Questions

Aspect	List of questions
General	What year were the studies published?
	Which academic communities are most active in health research using data from social media?
	Where are these studies originating and what is their geographical scope?
	What is the purpose for which social media data is being used in the research?
Topic	Which health topics are being studied?
SM Type	Which social media are used as a source of data?
Extract & analysis	What is the unit of analysis applied?
	How is the data from social media extracted and analysed and which proprietary tools were used?
Ethics	How are ethical considerations applied in the published research?

For peer review only

Stage 2: identifying relevant literature

Comprehensive scoping reviews aim to capture literature from a range of electronic databases, reference lists, and grey literature. As such, our approach will include:

- A systematic search of peer-reviewed studies using three health, informatics, business and cross-disciplinary electronic databases: PubMed, Scopus and Applied Social Sciences Index and Abstracts (ASSIA).
- 'Snowballing' from article reference lists [35] will be used to identify additional studies that may have not been indexed in the online research databases.
- Searching grey literature from the Internet using the most widely used search engine, Google [36]. The first 20 Google results yielded by each search string will be reviewed, as this search engine displays the results by relevance.

The search strategy for each of the databases was defined in consultation with a senior librarian. Our three electronic databases and one internet search engine takes account of time and funding constraints, although the sources targeted are likely to capture most of the relevant literature [37].

Formative searches:

Given the breadth and changing popularity of terms related to social media and social media mining, we undertook a formative analysis to understand those most likely to yield articles relevant to our review objectives. We first created an extensive list based on the search strings specified in a recent systematic review of social media in the context of e-government in public health [28], supplemented with terms captured from iterative online searches. The list included generic terms related to social media (e.g. digital, blog, social network, Web 2.0), named social media (e.g. Twitter, Facebook) types of social media mining (e.g. surveillance, scraping, listening, infoveillance) and analytics (e.g. netnography, social network analysis) [see Appendix 2 for the full list]. We then ran separate search queries for each term in PubMed, constructed as [*term*] *AND (health OR illness OR disease) AND human*. Searches yielding one or more hits were charted over time, to examine their changing frequency in the research literature. Out of the 72 terms tested in PubMed, 52 produced hits. The timeline was cross-referenced with key historical events, such as the introduction of new platforms or new methodologies. The results are shown in Figure 1.

*[**Figure 1.** Results of the search queries by year]*

As can be seen from Figure 1, 91% of relevant studies were published in the past 9 years (11,154 results between 2009-2017, vs. 1,048 between 1997-2008). Not surprisingly, the search term "social media" produced the highest number of results overall compared to other search queries. Regarding specific platforms, searches with Sixdegrees and Linkedin produced no results, compared with other social media such as Facebook (1152 hits, between 2008-2017), Twitter (939 hits, between 2009-2017), YouTube (351 hits, between 2008-2017), Patientslikeme (60 hits, between 2008-2017), and Instagram (40 hits, between 2012-2017). We also observed a time lag of four years between when Facebook, Patientslikeme and Twitter were launched in 2004, 2004 and 2006 respectively and the first research article was published, while the equivalent latency for YouTube and Instagram was two years. The individual search terms "surveillance" (665), "epidemiology" (581) and "ethnography" (110) produced the highest yields, compared with terms specific to digital research methods. With respect to the latter, temporal changes in the use of terms such as "infoveillance", "netnography", "social media listening", "social media analytics" and "social media mining" indicate the evolution of innovations and research perspectives, however including such specialist terms was not critical for identifying relevant studies.

As previously noted, a systematic search of three health, informatics, business and cross-disciplinary electronic databases - PubMed, Scopus and ASSIA – will be undertaken to identify relevant peer-reviewed studies. Below is the search query that will be used to interrogate these sources. This takes account of insights gathered during our formative analyses:

- Named social media [38], including health specific social media sites [39]. All searches that do not yield results will be excluded
- OR "web 2.0" OR "social media" OR "blog" OR "microblog" OR "wiki" OR "virtual world" OR "discussion forum" OR "online community"

For peer review only - http://bmjopen.bmj.com/site/about/guidelines.xhtml

- AND “surveillance” OR “epidemiology” OR “infoveillance” OR “ethnography” OR “netnography” OR “mining”
- AND (“health” OR “disease” OR “illness”)
- NOT “animal”

For Google searches the advanced search option will be used with English selected as the preferred language. All regions will be included and the search will be asked to find pages with the following search strings:

- “social media, surveillance, health”
- “social media, surveillance, disease”
- “social media, data mining, health”
- “social media, data mining, disease”
- “social media, epidemiology, health”
- “social media, epidemiology, disease”

Stage 3: study selection
One researcher will conduct the systematic search of electronic databases and grey literature. Studies will be selected after the abstracts and titles, identified via the electronic searches and “snowballing” technique, have been independently screened for relevance by two researchers based on the specified inclusion criteria.

Inclusion and exclusion criteria
Peer-reviewed journal articles and conference papers will be included where these describe empirical research using data from social media such as Twitter or Facebook, whether extracted or studied situ, using either manual or automated methods. Studies not in English, dissertations/theses, reports or abstracts, letters to the editor and feature articles and articles intended as marketing or advertising material will be excluded. A publication timeframe of 1997-2017 will be applied. See Box 1 for the inclusion and exclusion criteria for the scoping review.

Box 1. Article inclusion and exclusion criteria for the scoping review

**Inclusion**
Types of publication: Peer-reviewed academic research articles. Full conference papers<br>Language: English<br>Publication timeframe: 1997-2017<br>Types of research: Empirical studies using health-related data from social media, extracted or studied in situ, using both manual and automated methods.
**Exclusion**
Types of publication: Dissertations/theses; Reports or abstracts only; Letters to the editor; Marketing or advertising material; Reviews or editorials<br>Language: Not English<br>Types of research: Studies based on data from online sources other than social media (e.g. internet search histories, online news reports). Commercial research aimed at obtaining market intelligence or informing product promotion. Studies examining social media platforms, rather than using them as a source of data. Studies describing social media as a communication or broadcasting channel (e.g. for public health promotion).

Intermittent cross-checking by the second author will help to ensure the appropriate application of inclusion and exclusion criteria. In a further stage, the full-text of included articles will be independently assessed for eligibility by both authors and discrepancies resolved through discussion. Reasons for exclusion will be documented. These steps are described in Figure 2.

***[Figure 2.** Steps for identification, screening, eligibility and inclusion of studies in the scoping review]*

EndNote reference management software will be used to manage the records retrieved from searches. One reviewer will independently screen the generated citations with the help of EPPI-Reviewer 4 systematic review software, and undertake data extraction.

Stage 4: charting the data
The purpose of charting data in scoping reviews is to produce a descriptive summary of the results. For this stage, we have identified existing classification frameworks that correspond with the research questions listed previously. These will form the basis of our data charting form. The classification

For peer review only

frameworks consider the purpose for which social media data are being used in the research, the method of data extraction (including any automated data mining tools used), the analytical-interpretive approach used (including stated theoretical perspectives), the locus of the research by academic institution and geographical scope of the data, the academic discipline associated with the research and whether/how ethical issues or guidelines are considered. Each of these existing classification frameworks and taxonomies are considered in further detail in Table 3 in relation to the specific research question.

Table 3. Existing classification frameworks that correspond with the research questions

**Research question**	**Existing classification framework to be applied**	**How the classification framework will be applied**
What year were the studies published?	Not specified	The year that the eligible study was published will be captured.
Which academic communities are most active in conducting health research studies that use data from social media?	Scimago Journal Subject Areas [40] including 27 subject areas including medicine, computer science, health professions, business management and accounting as well as social sciences.	The journal in which eligible study is published, along with the first author's affiliation and source of funding for the study (if shown) will be classified according to the disciplinary taxonomy used by the Scimago Journal ranking portal. These categories are not mutually exclusive.
Where are these studies originating and what is their geographical scope?	World Bank Regional and Lending Groups for Countries includes seven regions and four lending groups [41]	The geographical location of the first author conducting the eligible study and the population sample analysed within the study will be classified according to the regional and lending groups provided by the World Bank for 2017. These categories are not mutually exclusive.
What is the purpose for which social media data are being used in the research?	Not specified	The purpose of the eligible study will be captured.
Which health topics are being studied?	The 19 health related topics which inform the World Health Organization's Sustainable Development Goals (SDG) [42]	The type of condition and health topic being studied in the eligible study will be classified according to the list of health related topics, which inform the WHO SDG. These categories are not mutually exclusive.
Which social media are used as a source of data?	Kaplan and Haenlein's eight types of social media [1]	The type of social media from which the data for the eligible study was sourced will be classified according to Kaplan and Haenlein's eight types of social media. The name of the social media will also be captured.
How large are the studies and what is the unit of analysis applied?	Not specified	The sample size and unit of analysis of the eligible study will be captured.
How is the data from social media extracted and analysed and which proprietary tools were used?	Several types of analysis may be performed on social media data, ranging from simple descriptive statistics to qualitative research to automated real-time analytics at scale as described by Batrinca and Treleaven [43]. These types of analysis have been described in terms of computational techniques, such as natural language processing, and purposive approaches such as news analytics, opinion mining, data scraping, sentiment analysis and text analytics to name but a few.	Batrinca and Treleaven taxonomy of social media analysis techniques will be used to guide our classification of the broad approaches and specific techniques demonstrated in the eligible studies. This taxonomy may be refined as a result of new insights emerging during data extraction. We will also capture the named type of analysis performed in the eligible study as well as any reference to proprietary tools used.
How are ethical considerations applied in the published research?	Conway's taxonomy of ethics concepts for the use of Twitter in public health surveillance and research [44] and can be applied across all manner of social media.	The application of ethical concepts in the eligible studies will be classified according to the ten high level categories identified by Conway. These categories are not mutually exclusive.

For peer review only

Stage 5: collating, summarizing and reporting the results
The extracted data will be tabulated, with rows relating to articles, columns to classification variables and cells containing the relevant information. Both frequency analysis and trend analysis will be used to chart the classified results.

Frequency analysis
Using frequency analysis, the counts and percentages of eligible studies will be calculated. Studies will be grouped based on the classification frameworks and taxonomies applied. The result of this analysis will be a map of eligible studies represented in bubble plot, graph or tabular form.

Trend analysis
Trend analysis will be used to present the changing frequency of research over the past 20 years, based on the aforementioned classification criteria. A map showing the characteristics of included studies will be presented using both static and multi-media visualization tools, such as an animated bubble plot or graph.

Public and Patient Involvement
No patients or members of the public were involved in the protocol design or exploratory analyses, nor do we plan to include them in the conduct of the scoping review. Our results are nevertheless likely to be of interest to citizens who use social media and our decision to examine how researchers report ethical considerations reflects this concern. Dissemination will include accessible summaries and graphics, which we intend to make available to the public via social media.

Ethics and dissemination
Although some scoping reviews include a consultation phase, this one focuses on published research and online sources already in the public domain. No ethics approval is therefore required.
The completed scoping review will be submitted for publication to a peer-reviewed, interdisciplinary Open Access journal, in addition to conferences on public health and digital research. Findings will be presented using both static and multimedia visualisation tools.

Competing interests: The authors declare no competing interests.

Contributorship: JT designed the protocol and undertook the exploratory searches, with input from CP. The manuscript was drafted by JT and edited by CP.

Funding: JT is a self-funded PhD student at the University of Edinburgh, supervised by CP. She also works as a management consultant for Ernst and Young Switzerland although her employers are not involved in this research. CP is a collaborator on three RCUK-funded research programmes associated with this study: The Administrative Data Research Centre for Scotland, ESRC, Grant number ES/L007487/1; The Science and Practice of Social Machines EPSRC, EP/ J017728/1 and the Farr Institute for Health Informatics Research (Scotland) MRC, Grant number MR/K007017/1. The funders had no role in study design, data collection and analysis, decision to publish, or preparation of the manuscript.

References
1. **Kaplan A**, **Haenlein M**: **Users of the world, unite! The challenges and opportunities of Social Media**. *Business Horizons* 2010, **53**(1):59-68.
2. **Complete History of Social Media: Then and Now** [https://smallbiztrends.com/2013/05/the-complete-history-of-social-media-infographic.html]
3. **Digital in 2017: Global Overview** [https://wearesocial.com/special-reports/digital-in-2017-global-overview]
4. **Social Media - Statistics and Facts** [https://www.statista.com/topics/1164/social-networks/]
5. **Current State of Social Media Research: From Practice to Theory #SMSociety15** [http://socialmedialab.ca/2015/current-state-of-social-media-research-from-practice-to-theory-part-1/]
6. **Social Media and Public Health Research** [https://bjerglund.files.wordpress.com/2012/11/final-social-media-and-public-health-research1.pdf]

For peer review only

7. Taylor J, Pagliari C: **Mining social media data: How are research sponsors and researchers addressing the ethical challenges?** *Research Ethics* 2017:1747016117738559.
8. Anstead N, O'Loughlin B: **Social Media Analysis and Public Opinion: The 2010 UK General Election**. *Journal of Computer-Mediated Communication* 2015, **20**(2):204-220.
9. **Social Media in Public Opinion Research: Report of the AAPOR Task Force on Emerging Technologies in Public Opinion Research** [https://www.aapor.org/AAPORKentico/AAPOR_Main/media/MainSiteFiles/AAPOR_Social_Media_Report_FNL.pdf]
10. Eysenbach G: **Infodemiology and Infoveillance: Framework for an Emerging Set of Public Health Informatics Methods to Analyze Search, Communication and Publication Behavior on the Internet**. *J Med Internet Res* 2009, **11**(1):e11.
11. Smith T: **The social media revolution**. *International Journal of Market Research* 2009, **51**(4):559.
12. Zafarani R, Ali Abbasi M, Liu H: **Social Media Mining: An Introduction**: Cambridge University Press; 2014.
13. **Social Analytics** [http://www.gartner.com/it-glossary/social-analytics]
14. Smart PR, Shadbolt NR: **Social Machines**. In: *Encyclopedia of Information Science and Technology*. edn. Edited by Khosrow-Pour M: IGI Global; 2014.
15. Kozinets RV: **Netnography**. In: *The International Encyclopedia of Digital Communication and Society*. edn.: John Wiley & Sons, Inc.; 2015.
16. Velasco E, Agheneza T, Denecke K, Kirchner G, Eckmanns TIM: **Social Media and Internet-Based Data in Global Systems for Public Health Surveillance: A Systematic Review**. *Milbank Quarterly* 2014, **92**(1):7-33.
17. Bernardo TM, Rajic A, Young I, Robiadek K, Pham MT, Funk JA: **Scoping review on search queries and social media for disease surveillance: a chronology of innovation**. *Journal of medical Internet research* 2013, **15**(7):e147.
18. Fung IC, Duke CH, Finch KC, Snook KR, Tseng PL, Hernandez AC, Gambhir M, Fu KW, Tse ZT: **Ebola virus disease and social media: A systematic review**. *American journal of infection control* 2016, **44**(12):1660-1671.
19. O'Shea J: **Digital disease detection: A systematic review of event-based internet biosurveillance systems**. *International journal of medical informatics* 2017, **101**:15-22.
20. Yan SJ, Chughtai AA, Macintyre CR: **Utility and potential of rapid epidemic intelligence from internet-based sources**. *International journal of infectious diseases : IJID : official publication of the International Society for Infectious Diseases* 2017, **63**:77-87.
21. Al-garadi MA, Khan MS, Varathan KD, Mujtaba G, Al-Kabsi AM: **Using online social networks to track a pandemic: A systematic review**. *Journal of Biomedical Informatics* 2016, **62**:1-11.
22. **A systematic literature review on event-based public health surveillance systems** [http://edoc.sub.uni-hamburg.de/haw/volltexte/2012/1614/pdf/lsab12_27.pdf]
23. Guy S, Ratzki-Leewing A, Bahati R, Gwadry-Sridhar F: **Social Media: A Systematic Review to Understand the Evidence and Application in Infodemiology**. In: *Electronic Healthcare: 2012// 2012; Berlin, Heidelberg*: Springer Berlin Heidelberg; 2012: 1-8.
24. Verhoef LM, Van de Belt TH, Engelen LJLPG, Schoonhoven L, Kool RB: **Social media and rating sites as tools to understanding quality of care: a scoping review**. *Journal of medical Internet research* 2014, **16**(2):e56.
25. Emmert M, Sander U, Pisch F: **Eight Questions About Physician-Rating Websites: A Systematic Review**. *J Med Internet Res* 2013, **15**(2):e24.
26. Golder S, Norman G, Loke YK: **Systematic review on the prevalence, frequency and comparative value of adverse events data in social media**. *British journal of clinical pharmacology* 2015, **80**(4):878-888.
27. Kazemi DM, Borsari B, Levine MJ, Dooley B: **Systematic review of surveillance by social media platforms for illicit drug use**. *Journal of public health (Oxford, England)* 2017:1-14.
28. Tursunbayeva A, Franco M, Pagliari C: **Use of social media for e-Government in the public health sector: A systematic review of published studies**. *Government Information Quarterly* 2017, **34**(2):270-282.
29. Patel R, Chang T, Greysen SR, Chopra V: **Social Media Use in Chronic Disease: A Systematic Review and Novel Taxonomy**. *The American Journal of Medicine*, **128**(12):1335-1350.
30. Golder S, Ahmed S, Norman G, Booth A: **Attitudes Toward the Ethics of Research Using Social Media: A Systematic Review**. *Journal of medical Internet research* 2017, **19**(6):e195.

31. Arksey H, O'Malley L: **Scoping studies: towards a methodological framework**. *International Journal of Social Research Methodology* 2005, **8**(1):19-32.
32. Levac D, Colquhoun H, O'Brien KK: **Scoping studies: advancing the methodology**. *Implementation Science : IS* 2010, **5**:69-69.
33. **Chapter 11: Scoping Reviews.** [Available from https://reviewersmanual.joannabriggs.org/]
34. Armstrong R, Hall Bj Fau - Doyle J, Doyle J Fau - Waters E, Waters E: **Cochrane Update. 'Scoping the scope' of a cochrane review**. (1741-3850 (Electronic)).
35. Horsley T, Dingwall O, Sampson M: **Checking reference lists to find additional studies for systematic reviews**. *Cochrane Database of Systematic Reviews* 2011(8).
36. **Search Engine Market Share** [https://netmarketshare.com/search-engine-market-share.aspx?options=%7B%22filter%22%3A%7B%22%24and%22%3A%5B%7B%22deviceType%22%3A%7B%22%24in%22%3A%5B%22Desktop%2Flaptop%22%5D%7D%7D%5D%7D%2C%22dateLabel%22%3A%22Trend%22%2C%22attributes%22%3A%22share%22%2C%22group%22%3A%22searchEngine%22%2C%22sort%22%3A%7B%22share%22%3A-1%7D%2C%22id%22%3A%22searchEnginesDesktop%22%2C%22dateInterval%22%3A%22Monthly%22%2C%22dateStart%22%3A%222017-03%22%2C%22dateEnd%22%3A%222018-02%22%2C%22segments%22%3A%22-1000%22%7D]
37. Lam MT, McDiarmid M: **Increasing number of databases searched in systematic reviews and meta-analyses between 1994 and 2014**. *Journal of the Medical Library Association : JMLA* 2016, **104**(4):284-289.
38. **List of social networking websites** [http://en.wikipedia.org/wiki/List_of_social_networking_websites]
39. **Top 25 Health and Medical Social Media Sites** [http://mastersinhospitaladministration.com/2011/top-25-health-and-medical-social-media-sites/]
40. **Scimago Journal & Country Rank** [http://www.scimagojr.com/journalrank.php]
41. **World Bank Country and Lending Groups** [https://datahelpdesk.worldbank.org/knowledgebase/articles/906519-world-bank-country-and-lending-groups]
42. **World Health Statistics 2016 data visualizations dashboard: Monitoring health for SDGs** [http://apps.who.int/gho/data/node.sdg.tp-1?lang=en]
43. Batrinca B, Treleaven PC: **Social media analytics: a survey of techniques, tools and platforms**. *AI & SOCIETY* 2015, **30**(1):89-116.
44. Conway M: **Ethical Issues in Using Twitter for Public Health Surveillance and Research: Developing a Taxonomy of Ethical Concepts From the Research Literature**. *J Med Internet Res* 2014, **16**(12):e290.

For peer review only

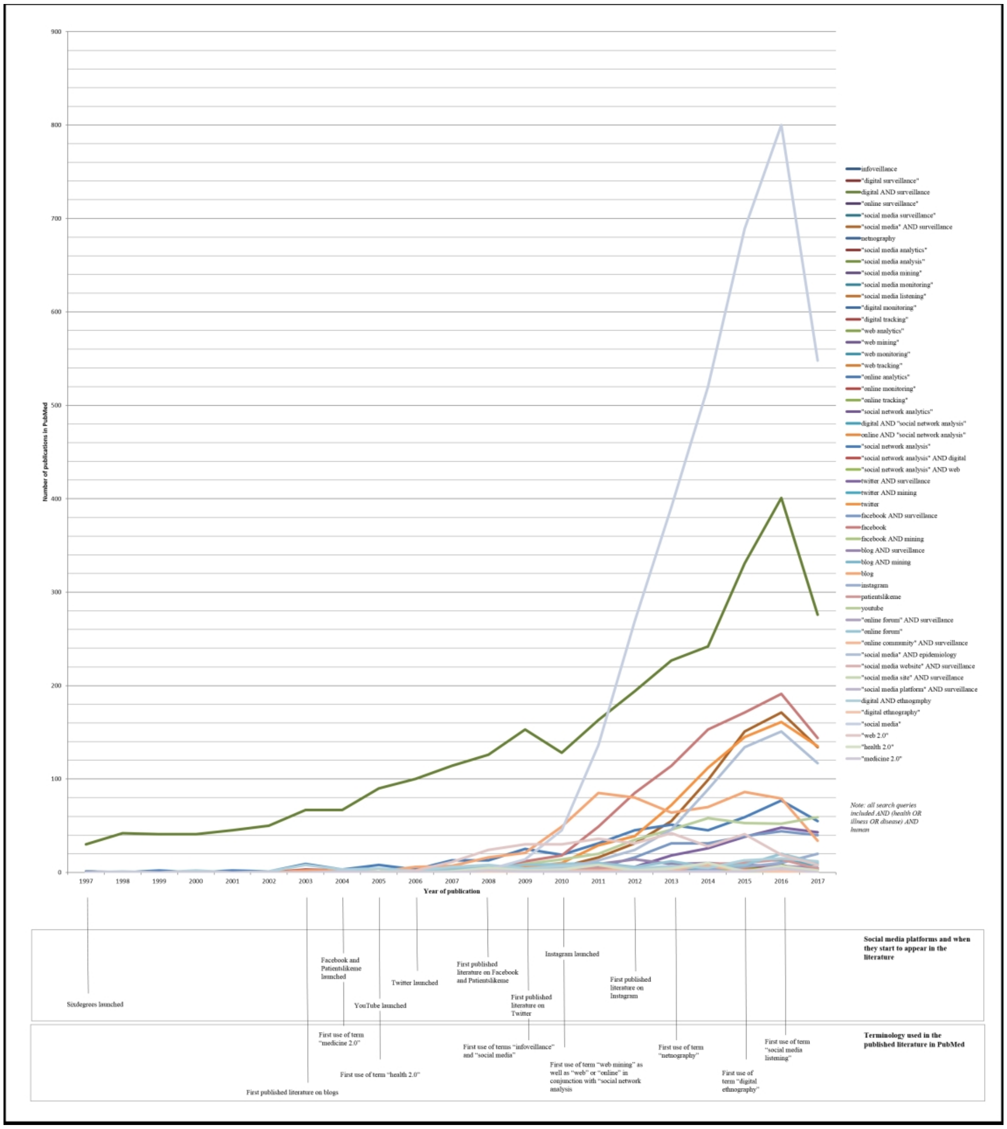

Figure 1. Results of the search queries by year

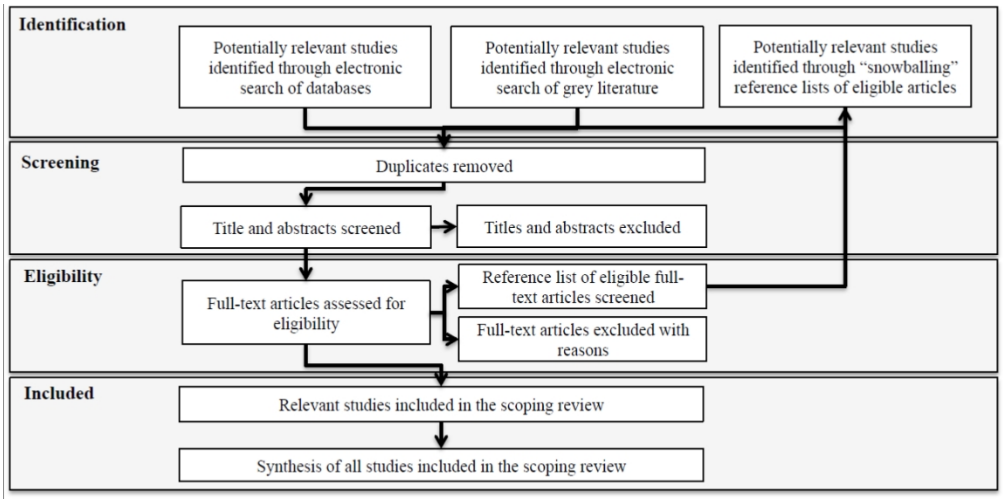

Figure 2. Steps for identification, screening, eligibility and inclusion of studies in the scoping review

Appendix 1. Scoping and systematic reviews on the secondary use of data from social media

	Systematic or Quasi-Systematic Scoping Review	Review Aim/Objective/Main Focus	Number of Databases Used	Key Findings
1	Velasco, E., Agheneza, T., Denecke, K., Kirchner, G., Eckmanns, T.<br>Social media and internet-based data in global systems for public health surveillance: a systematic review. Milbank Q. 2014; 92(1): 7-33	To explore how useful are data from social media and the Internet, and what is the potential to enhance surveillance.	3	• No event-based surveillance systems currently used in national surveillance programs<br>• Existing systems have limitations<br>• There is a need for further development of automated technologies that monitor health-related information on the Internet<br>• The dissemination to health authorities of new information about health events is not always efficient and could be improved.<br>• No comprehensive evaluations show whether event-based surveillance systems have been integrated into actual epidemiological work during real-time health events.
2	Golder, S., Norman, G., Loke, Y.K.<br>Systematic review on the prevalence, frequency and comparative value of adverse events data in social media<br>British Journal of Clinical Pharmacology. 2015; 80(4); 878-88	To summarize the prevalence, frequency and comparative value of information on the adverse events of healthcare interventions from user comments and videos in social media.	16 databases and 2 internet search engines	• The prevalence of adverse events reports on social media varies from 0.2% to 8% of posts.<br>• 'Mild' and symptom-related adverse events are over-represented in social media and laboratory test abnormalities and 'serious' adverse events are under- represented compared with other data sources.<br>• The question as to whether searching social media for adverse events data is a valuable use of resources, resulting in improved patient outcomes remains unanswered.
3	Verhoef, L.M., Van de Belt, T.H.,<br>Engelen, L.J.L.P.G., Schoonhoven, L., Kool, R.B.<br>Social media and rating sites as tools to understanding quality of care: a scoping review<br>J Med Internet Res. 2014; 16(2); e56	To explore the relationship between data from social media and quality of care.	4	• There is a relationship between information on social media and quality of health care<br>• Drawbacks exist, since rating is anonymous and vulnerable to fraud. They are often based on only a few reviews and are predominantly positive.<br>• People providing feedback on health care via social media are presumably not always representative for the patient population.
4	Bernardo, T.M., Rajic, A., Young, I., Robiadek, K., Pham, M.T., Funk, J.A.<br>Scoping review on search queries and social media for disease surveillance: a chronology of innovation<br>J Med Internet Res. 2013; 15(7); e147	To assess the current state of knowledge regarding the use of search queries and social media for disease surveillance in order to inform future work on early detection and more effective mitigation of the effects of foodborne illness.	1 database and 2 internet search engines	• The use of search queries and social media for disease surveillance are relatively recent phenomena (first reported in 2006).<br>• Both the tools themselves and the methodologies for exploiting them are evolving over time.<br>• While their accuracy, speed, and cost compare favorably with existing surveillance systems, the primary challenge is to refine the data signal by reducing surrounding noise.
5	Fung, Tse, Cheung, Miu, & Fu. (2014). Ebola and the social media. The Lancet,	To systematically reviewed existing research pertinent to Ebola virus disease and social media, especially	6	• Included 7 articles on Twitter, 1 on Facebook, 3 on YouTube, 1 on Instagram & Flickr.

For peer review only

	Systematic or Quasi-Systematic Scoping Review	Review Aim/Objective/Main Focus	Number of Databases Used	Key Findings
	384(9961), 2207.	to identify the research questions and the methods used to collect and analyze social media.		• 11 articles examined Ebola posts' content themes, meta-data or user characteristics.<br>• One article examined how Ebola-related news videos influenced Twitter traffic.
6	Golder S, Ahmed S, Norman G, Booth A Attitudes Toward the Ethics of Research Using Social Media: A Systematic Review J Med Internet Res 2017;19(6):e195	To ascertain attitudes on the ethical considerations of using social media as a data source for research as expressed by social media users and researchers.	16 data bases and 2 internet search engines	• Attitudes varied from overly positive with people expressing the views about the essential nature of such research for the public good, to very concerned with views that social media research should not happen.<br>• Underlying reasons for this variation related to issues such as the purpose and quality of the research, the researcher affiliation, and the potential harms.<br>• The methods used to conduct the research were also important.<br>• Many respondents were positive about social media research while adding caveats such as the need for informed consent or use restricted to public platforms only.
7	Kazemi, D., Borsari, B., Levine, M., & Dooley, B. (2017). Systematic review of surveillance by social media platforms for illicit drug use. Journal of Public Health (Oxford, England), 1-14.	To review the ability of social media to better recognize illicit drug use trends was addressed.	9	Selected studies used a range of social media tools/applications, including message boards, Twitter and blog/forums/platform discussions.
8	O'Shea, Jesse. (2017). Digital disease detection: A systematic review of event-based internet biosurveillance systems. International Journal of Medical Informatics, 101, 15-22.	To update the current state of knowledge on event-based Internet biosurveillance systems by identifying all systems, including current functionality, with hopes to aid decision makers with whether to incorporate new methods into comprehensive programmes of surveillance.	3	• Each system uses different innovative technology and data sources to gather data, process, and disseminate data to detect infectious disease outbreaks.
9	Yan, Chughtai, & Macintyre. (2017). Utility and potential of rapid epidemic intelligence from internet-based sources. International Journal of Infectious Diseases, 63, 77-87.	To identify and summarise the types of Internet-based surveillance methods studied in recent literature. A secondary aim was to identify and summarise the timeliness and accuracy outcomes of Internet-based methods described in literature.	5	• Studies used search queries, social media posts and approaches derived from existing Internet-based systems for early epidemic alerts and real-time monitoring.<br>• Most studies noted improved timeliness compared to official reporting<br>• Internet-based methods showed variable correlation strength with official datasets, with some methods showing reasonable accuracy.
10	Al-Garadi, Khan, Varathan, Mujtaba, & Al-Kabsi. (2016). Using online social networks to track a pandemic: A systematic review. Journal of Biomedical Informatics, 62, 1-11.	To conduct a systematic review of the use of online social networks to track a pandemic.	5	• Identified the extent of using online social networks to track a pandemic.<br>• Online social networks contain significant information to track a pandemic almost in real time.<br>• There are challenges and future implications.
11	Agheneza T. A Systematic Review of Event-Based Public Health Surveillance Systems. Hamburg: Faculty	To identify all the event-based surveillance systems that exist and where they are based.	3	• 13 event-based systems were identified, and with 10 of the 13 systems evaluated.<br>• N. America is the leading

For peer review only

	Systematic or Quasi-Systematic Scoping Review	Review Aim/Objective/Main Focus	Number of Databases Used	Key Findings
	of Life Sciences, Hamburg University of Applied Sciences; 2011.			continent with about 77 % of the event-based systems followed by Europe and lastly Asia. No system was found in Africa, Australia and S. America.
12	Emmert M, Sander U, Pisch F. Eight questions about physician-rating websites: a systematic review. J Med Internet Res. 2013;15(2):e24.	To identify frequently discussed issues about physician-rating websites.	7	• The current usage of physician-rating websites is still low but is increasing.<br>• International data show that 1 out of 6 physicians has been rated, and approximately 90% of all ratings on physician-rating websites were positive.<br>• Could not find any evidence of "doctor-bashing".<br>• Physicians should not ignore these websites, but rather, monitor the information available and use it for internal and external purposes.
13	Guy S, Ratzki-Leewing A, Bahati R, Gwadry-Sridhar F. Social media: A systematic review to understand the evidence and application in infodemiology. Lect Notes Inst Comput Sci Soc Informatics Telecommun Eng. 2012;91:1–8.	To systematically review the literature utilizing social media as a source for disease prediction and surveillance.	2	• Open-source micro-blogging sites can be used for influenza-like-illness monitoring.
14	Tursunbayeva, Franco, & Pagliari. (2017). Use of social media for e-Government in the public health sector: A systematic review of published studies. Government Information Quarterly, 34(2), 270-282.	To investigate the adoption and use of social media by public health organizations	4	• Evidence-base is mostly descriptive, unidisciplinary and lacks the theoretical depth seen in other branches of e-Government research.<br>• The lack of robust evidence makes it difficult to draw conclusions about the effectiveness of these approaches in the public health sector
15	Patel, Chang, Greysen, & Chopra. (2015). Social Media Use in Chronic Disease: A Systematic Review and Novel Taxonomy. The American Journal of Medicine, 128(12), 1335-1350.	To evaluate clinical outcomes from applications of contemporary social media in chronic disease; to develop a conceptual taxonomy to categorize, summarize, and then analyze the current evidence base; and to suggest a framework for future studies	1	• The overall impact of social media on chronic disease was variable.<br>• Among studies that showed benefit, most used either Facebook or blogs, and were based within the domain of support.

For peer review only

Appendix 2. Total number of publications by search query

**Search query**	**Total number of publications from 1997-2017**
"social media" AND (health OR illness OR disease) AND human	3425
digital AND surveillance AND (health OR illness OR disease) AND human	2935
facebook AND (health OR illness OR disease) AND human	939
twitter AND (health OR illness OR disease) AND human	709
"social media" AND surveillance AND (health OR illness OR disease) AND human	665
blog AND (health OR illness OR disease) AND human	602
"social media" AND epidemiology AND (disease OR health OR illness) AND human	581
"social network analysis" AND (disease OR health OR illness) AND human	462
youtube AND (health OR illness OR disease) AND human	351
"web 2.0" AND (health OR illness OR disease) AND human	312
facebook AND surveillance AND (health OR illness OR disease) AND human	206
twitter AND surveillance AND (health OR illness OR disease) AND human	187
digital AND ethnography AND (health OR illness OR disease) AND human	110
"online forum" AND (health OR illness OR disease) AND human	103
blog AND surveillance AND (health OR illness OR disease) AND human	76
patientslikeme AND (health OR illness OR disease) AND human	60
"online monitoring" AND (disease OR health OR illness) AND human	48
"health 2.0" AND (health OR illness OR disease) AND human	46
twitter AND mining AND (health OR illness OR disease) AND human	43
instagram AND (health OR illness OR disease) AND human	40
online AND "social network analysis" AND (disease OR health OR illness) AND human	29
"online forum" AND surveillance AND (health OR illness OR disease) AND human	25
"online community" AND surveillance AND (health OR illness OR disease) AND human	25
infoveillance AND (disease OR health OR illness) AND human	24
"online surveillance" AND (disease OR health OR illness) AND human	23
"social network analysis" AND web AND (disease OR health OR illness) AND human	23
"digital surveillance" AND (disease OR health OR illness) AND human	16
netnography AND (disease OR health OR illness) AND human	14
"medicine 2.0" AND (health OR illness OR disease) AND human	14
"social media analysis" AND (disease OR health OR illness) AND human	12
"online tracking" AND (disease OR health OR illness) AND human	12
"web analytics" AND (disease OR health OR illness) AND human	10
"social media analytics" AND (disease OR health OR illness) AND human	9
"social media monitoring" AND (disease OR health OR illness) AND human	9
blog AND mining AND (health OR illness OR disease) AND human	9
"web mining" AND (disease OR health OR illness) AND human	8
"digital monitoring" AND (disease OR health OR illness) AND human	7
facebook AND mining AND (health OR illness OR disease) AND human	7
"social media mining" AND (disease OR health OR illness) AND human	6
"web monitoring" AND (disease OR health OR illness) AND human	6

For peer review only

Search query	Total number of publications from 1997-2017
"social media platform" AND surveillance AND (health OR illness OR disease) AND human	6
"social media surveillance" AND (disease OR health OR illness) AND human	4
"web tracking" AND (disease OR health OR illness) AND human	4
digital AND "social network analysis" AND (disease OR health OR illness) AND human	4
"social network analysis" AND digital AND (disease OR health OR illness) AND human	4
"digital ethnography" AND (health OR illness OR disease) AND human	4
"digital tracking" AND (disease OR health OR illness) AND human	3
"online analytics" AND (disease OR health OR illness) AND human	3
"social media site" AND surveillance AND (health OR illness OR disease) AND human	3
"social media listening" AND (disease OR health OR illness) AND human	2
"social network analytics" AND (disease OR health OR illness) AND human	2
"social media website" AND surveillance AND (health OR illness OR disease) AND human	2
"web surveillance" AND (disease OR health OR illness) AND human	0
"social network surveillance" AND (disease OR health OR illness) AND human	0
"social media observing" AND (disease OR health OR illness) AND human	0
"social media tracking" AND (disease OR health OR illness) AND human	0
"digital observing" AND (disease OR health OR illness) AND human	0
"digital analytics" AND (disease OR health OR illness) AND human	0
"digital mining" AND (disease OR health OR illness) AND human	0
"digital listening" AND (disease OR health OR illness) AND human	0
"web observing" AND (disease OR health OR illness) AND human	0
"web listening" AND (disease OR health OR illness) AND human	0
"online observing" AND (disease OR health OR illness) AND human	0
"online mining" AND (disease OR health OR illness) AND human	0
"online listening" AND (disease OR health OR illness) AND human	0
"social network observing" AND (disease OR health OR illness) AND human	0
"social network mining" AND (disease OR health OR illness) AND human	0
"social network monitoring" AND (disease OR health OR illness) AND human	0
"social network listening" AND (disease OR health OR illness) AND human	0
"social network tracking" AND (disease OR health OR illness) AND human	0
linkedin AND (health OR illness OR disease) AND human	0
"sixdegrees" AND (health OR illness OR disease) AND human	0

For peer review only

BMJ Open

A Comprehensive Scoping Review of Health Research Using Social Media Data (Protocol)

Journal:	*BMJ Open*
Manuscript ID	bmjopen-2018-022931.R1
Article Type:	Protocol
Date Submitted by the Author:	10-Jul-2018
Complete List of Authors:	Taylor, Joanna; University of Edinburgh, eHealth Research Group, Usher Institute of Population Health Sciences and Informatics; Ernst and Young<br><br>Pagliari, Claudia; University of Edinburgh, eHealth Research Group, Usher Institute for Population Health Sciences
**Primary Subject Heading**:	Health informatics
Secondary Subject Heading:	Public health
Keywords:	Social Media, eHealth, Digital Research, Public Health Informatics, Data Science, Digital Health

SCHOLARONE™
Manuscripts

A Comprehensive Scoping Review of Health Research Using Social Media Data (Protocol)

Joanna Taylor and Claudia Pagliari

Corresponding Author: Claudia Pagliari, eHealth Research Group, Usher Institute for Population Health Sciences and Informatics, University of Edinburgh, Medical School, Teviot Place, Edinburgh EH8 9AG, UK.
Email: claudia.pagliari@ed.ac.uk
Tel: 0131 6509464
Fax: 0131 6509119

Joanna Taylor
Usher Institute for Population Health Sciences and Informatics, The University of Edinburgh, UK
Ernst and Young Ltd, Switzerland

Claudia Pagliari
Usher Institute for Population Health Sciences and Informatics, The University of Edinburgh, UK
claudia.pagliari@ed.ac.uk

Keywords: Social Media [MeSH]**, eHealth, Digital Research, Public Health Informatics** [MeSH]**, Data Science**

Word count: 2823 (excluding tables)

For peer review only

Abstract

Introduction: The rising popularity of social media, since their inception around 20 years ago, has been echoed in the growth of health-related research using data derived from them. This has created a demand for literature reviews to synthesise this emerging evidence-base and inform future activities. Existing reviews tend to be narrow in scope, with limited consideration of the different types of data, analytical methods and ethical issues involved. There has also been a tendency for research to be siloed within different academic communities (e.g. computer science, public health), hindering knowledge translation. To address these limitations, we will undertake a comprehensive scoping review, to systematically capture the broad corpus of published, health-related research based on social media data. Here we present the review protocol and the pilot analyses used to inform it.

Methods: A version of Arksey and O'Malley's five-stage scoping review framework will be followed: (1) identifying the research question; (2) identifying the relevant literature; (3) selecting the studies; (4) charting the data; and (5) collating, summarizing and reporting the results. To inform the search strategy we developed an inclusive list of keyword combinations related to social media, health and relevant methodologies. The frequency and variability of terms were charted over time and cross referenced with significant events, such as the advent of Twitter. Five leading health, informatics, business and cross-disciplinary databases will be searched: PubMed, Scopus, ACM, IEEE and Applied Social Sciences Index and Abstracts, alongside the Google search engine. There will be no restriction by date.

Ethics and dissemination: The review focuses on published research in the public domain therefore no ethics approval is required. The completed review will be submitted for publication to a peer-reviewed, interdisciplinary Open Access journal, and conferences on public health and digital research.

Article Summary

Strengths and limitations of this study

- The proposed scoping review addresses the need for a comprehensive, cross-disciplinary synthesis of health-related research using social media data.
- A 5-stage scoping review protocol was developed, which was informed by an exploratory analysis of existing reviews, terminologies and published taxonomies.
- This revealed the changing usage of relevant terms over time, the types of keyword combinations most likely to yield eligible studies, and the time-lag between the launch of new social media platforms and published research using data derived from them.
- The findings of the scoping review will be communicated using both static and multi-media visualisation tools.

For peer review only

Introduction - Context and coverage of the existing review literature

Social media are online, often mobile, platforms that support the creation and exchange of user-generated content [1]. They include generic platforms for networking, information sharing and collaboration (e.g. Facebook, Twitter, YouTube, Linkedin) and online forums aimed at specific communities (e.g. Patientslikeme, Mumsnet). The first modern day social media platform, sixdegrees, was launched in 1997 and allowed users to connect with friends and family through sending messages and posts on bulletin boards [2]. Since then the use of social media has become increasingly common [3] with an estimated 2.5 billion of the global population estimated to be using them as of 2017 [4].

As social media have become an integral part of people's lives, research focusing on social media has also evolved and is taking place in a number of domains. A bibliometric content analysis of key words that appeared in the abstracts of 14,500 journal articles and conference papers identified five main areas in which a high volume of research already exists. This includes research related to health, educational uses of social media, computing and computer science methods, business, organizational and marketing topics, and political and social engagement [5].

Uses of social media in the context of research can be described according to the broad categories identified by Bjerglund-Andersen and Söderqvist (dissemination, discussion/networking, public engagement, teaching, research/data collection) [6]. Taylor and Pagliari (2017) [7] recently separated the last of these into social media as a *source of data* for research and as a tool for *the conduct of research*. These 'secondary uses' of social media data include analyses of trends, associations and sentiments in users' postings, as well as interactions and networks [8, 9].

This new source of 'big data' has triggered scientific developments in a number of areas, including health. The term "infoveillance" was coined by Eysenbach in 2009 to describe the automated harvesting and analysis of internet searches and social media postings as an alternative approach for health and disease surveillance [10]. Other terms have since been introduced across academia and industry to describe the use of such data for gleaning insights about behavioural trends, determining the impact of interventions or predicting future events. These include "social media listening" [11], "social media mining" [12], "social analytics" [13], "social machines" [14] and "netnography" [15], to name but a few.

Based on an iterative search using PubMed we identified an existing corpus of 15 systematic, quasi-systematic and scoping reviews on the secondary use of social media data for health research, examples of which are summarized in Table 1 [see Appendix 1 for the full list, summarised according to their aims, number of databases and findings]. Amongst these are reviews on approaches to health surveillance [16-23] quality of healthcare services [24, 25], adverse event monitoring [26], illicit drug use [27], eGovernment [28], chronic conditions [29] and ethics [30].

Table 1. Existing systematic, quasi-systematic or scoping reviews indexed in PubMed.

Topic	Examples of Systematic, Quasi-systematic or Scoping Reviews
Disease surveillance	Social media and internet-based data in global systems for public health surveillance: a systematic review [16]
	Scoping review on search queries and social media for disease surveillance: a chronology of innovation [17]
	Ebola and the social media. [18]
	Digital disease detection: A systematic review of event-based internet biosurveillance systems. [19]
	Utility and potential of rapid epidemic intelligence from internet-based sources. [20]
	Using online social networks to track a pandemic: A systematic review. [21]
	A Systematic Review of Event-Based Public Health Surveillance Systems. [22]
	Social media: A systematic review to understand the evidence and application in infodemiology. [23]
Adverse event monitoring	Systematic review on the prevalence, frequency and comparative value of adverse events data in social media [26]
Quality of healthcare services	Social media and rating sites as tools to understanding quality of care: a scoping review [24]
	Eight questions about physician-rating websites: a systematic review. [25]
Illicit drug use	Systematic review of surveillance by social media platforms for illicit drug use. [27]
eGovernment	Use of social media for e-Government in the public health sector: A systematic review of published studies. [28]
Chronic disease	Social Media Use in Chronic Disease: A Systematic Review and Novel Taxonomy [29]

For peer review only

Ethics	Attitudes Toward the Ethics of Research Using Social Media: A Systematic Review [30]

A 2013 scoping review by Bernardo et al. on the use of internet search queries and social media for disease surveillance identified the first study as has having been published in 2006 and described how techniques for exploiting this data are evolving to increase the accuracy of signal detection [17]. Although insightful in understanding the chronology of this type of innovation, the review searched only 1 database and focused on the surveillance of foodborne illness. A 2014 systematic review by Velasco et al. on the impact and use of data from social media for public health surveillance went further, concluding that existing surveillance systems are limited and there is a need for automated technologies to monitor health related information on the Internet, although it did not specify which types of systems were analysed [16]. O'Shea et al.'s 2017 systematic review of event-based internet biosurveillance systems describes the wide variety of technologies and data sources for gathering, processing and disseminating data to detect infectious disease outbreaks [19]. These reviews, alongside the others listed, focus predominantly on infectious disease. A systematic review on social media for chronic disease exists [29] which focuses on understanding the clinical outcomes associated with using such technologies for patient support, education and disease management across different conditions. However this is limited by the inclusion of only database, and did not examine the type of methods or tools used to extract and analyse social media data, the academic discipline and setting of the research or the ethical issues considered. With respect to the latter, a recent systematic review by Golder et al., analysed studies reporting attitudes to research using social media data. This revealed wide variation in attitudes, from the very positive to the very concerned, depending on the purpose and quality of the research, researcher affiliation, the potential for harm and the methods used [30]. Although it used an impressive 16 databases, this review did not examine regional, disciplinary, condition or health topic specific variations and the authors note that the demographic characteristics of respondents was unclear in most studies. As noted in a recent review of UK research ethics guidelines and published health research, growing public awareness of the misuse of social media for marketing and algorithmic prediction, are forcing policymakers to look more closely at this issue [7].

In light of these gaps, we set out to undertake a comprehensive scoping review aimed at capturing and profiling a broad corpus of published multi-disciplinary research in which data obtained from social media have been used to monitor, understand or evaluate aspects of health and disease. In this paper, we describe the formal protocol for the comprehensive scoping review, alongside the preliminary analyses undertaken to inform each stage.

Methods and analysis

Scoping reviews are a type of quasi-systematic review that are increasingly used for understanding research on emerging innovations, which may be poorly indexed, distributed across published and grey literature or located in different academic disciplines [31-33]. They typically progress in five key stages (1) identifying the research question; (2) identifying the relevant studies; (3) selecting the studies; (4) charting the data; and (5) collating, summarizing and reporting the results. Developing an *a priori* review protocol can be useful for managing this complexity, while formative research can aid the design of such protocols by identifying relevant terminologies, topics and evidence sources. Scoping reviews are mainly aimed at mapping the evidence landscape rather than establishing the effectiveness of particular interventions and typically do not involve critical appraisal of study methodology or detailed extraction of outcomes data [34].

Stage 1: identifying the research question

In addition to the overarching review objective articulated in the introduction, several specific questions will be used to guide our analysis of existing research evidence, as listed in Table 2.

Table 2. Research Questions

Aspect	List of questions
General	How frequently has this type of research been published in different years?
	What terms are being used to describe the nature of this research?
	Which academic communities are most active in health research using data from social media?
	Where are study authors located, according to their affiliation?
	What is the geographical scope of the social media data analysed in these studies?
	For what purposes are social media data being used in this research?

For peer review only - http://bmjopen.bmj.com/site/about/guidelines.xhtml

Topic	Which health topics are being studied?
SM Type	Which social media platforms or sites are being used as sources of data?
Extract & analysis	What units of analysis are being applied?
	How are data from social media being extracted and analysed and which proprietary tools are being used?
Ethics	How are ethical considerations applied in the published research?

Stage 2: identifying relevant literature

Comprehensive scoping reviews aim to capture literature from a range of electronic databases, reference lists, and grey literature. As such, our approach will include:

- A systematic search of peer-reviewed studies using five health, informatics, business and cross-disciplinary electronic databases: PubMed, Scopus, Association of Computer Machinery (ACM), Institute of Electrical and Electronics Engineers (IEEE) and Applied Social Sciences Index and Abstracts (ASSIA).
- 'Snowballing' from article reference lists [35] will be used to identify additional studies that may have not been indexed in the online research databases.
- Searching grey literature from the Internet using the most widely used search engine, Google [36]. The first 20 Google results yielded by each search string will be reviewed, as this search engine displays the results by relevance.

The search strategy for each of the databases was defined in consultation with a senior librarian. Our five electronic databases and one internet search engine takes account of time and funding constraints, although the sources targeted are likely to capture most of the relevant literature [37].

Formative searches:

Given the breadth and changing popularity of terms related to social media and social media mining, we undertook a formative analysis to understand those most likely to yield articles relevant to our review objectives. We first created an extensive list based on the search strings specified in a recent systematic review of social media in the context of e-government in public health [28], supplemented with terms captured from iterative online searches. The list included generic terms related to social media (e.g. digital, blog, social network, Web 2.0), named social media (e.g. Twitter, Facebook), types of social media mining (e.g. surveillance, scraping, listening, infoveillance) and analytics (e.g. netnography, social network analysis) [see Appendix 2 for the full list]. We then ran separate search queries for each term in PubMed, constructed as [*term*] *AND (health OR illness OR disease) AND human*. Searches yielding one or more hits were charted over time, to examine their changing frequency in the research literature. Out of the 72 terms tested in PubMed, 52 produced hits. The timeline was cross-referenced with key historical events, such as the introduction of new platforms or new methodologies. The results are shown in Figure 1.

*[**Figure 1.** Results of the search queries by year]*

As can be seen from Figure 1, 91% of relevant studies were published in the past 9 years (11,154 results between 2009-2017, vs. 1,048 between 1997-2008). Not surprisingly, the search term "social media" produced the highest number of results overall compared to other search queries. Regarding specific platforms, searches with sixdegrees and Linkedin produced no results, compared with other social media such as Facebook (1152 hits, between 2008-2017), Twitter (939 hits, between 2009-2017), YouTube (351 hits, between 2008-2017), Patientslikeme (60 hits, between 2008-2017), and Instagram (40 hits, between 2012-2017). We also observed a time lag of four years between when Facebook, Patientslikeme and Twitter were launched in 2004, 2004 and 2006 respectively and the first research article was published, while the equivalent latency for YouTube and Instagram was two years. The individual search terms "surveillance" (665), "epidemiology" (581) and "ethnography" (110) produced the highest yields, compared with terms specific to digital research methods. With respect to the latter, temporal changes in the use of terms such as "infoveillance", "netnography", "social media listening", "social media analytics" and "social media mining" indicate the evolution of innovations and research perspectives, however including such specialist terms was not critical for identifying relevant studies.

As previously noted, a systematic search of five health, informatics, business and cross-disciplinary

For peer review only

electronic databases - PubMed, Scopus, ACM, IEEE and ASSIA – will be undertaken to identify relevant peer-reviewed studies. Below is the search query that will be used to interrogate these sources. This takes account of insights gathered during our formative analyses:

- Named social media [38], including health specific social media sites [39]. All searches that do not yield results will be excluded
- OR "web 2.0" OR "social media" OR "blog" OR "microblog" OR "wiki" OR "virtual world" OR "discussion forum" OR "online community"
- AND "surveillance" OR "epidemiology" OR "infoveillance" OR "ethnography" OR "netnography" OR "mining"
- AND ("health" OR "disease" OR "illness")
- NOT "animal"

For Google searches the advanced search option will be used with English selected as the preferred language. All regions will be included and the following search strings will be used:

- "social media, surveillance, health, NOT animal"
- "social media, surveillance, disease, NOT animal"
- "social media, data mining, health, NOT animal"
- "social media, data mining, disease, NOT animal"
- "social media, epidemiology, health, NOT animal"
- "social media, epidemiology, disease, NOT animal"

Stage 3: study selection

One researcher will conduct the systematic search of electronic databases and grey literature. Studies will be selected after the abstracts and titles, identified via the electronic searches and "snowballing" technique, have been independently screened for relevance by two researchers based on the specified inclusion criteria.

Inclusion and exclusion criteria

Peer-reviewed journal articles and conference papers will be included where these describe empirical research using data from social media such as Twitter or Facebook, whether extracted or studied situ, using either manual or automated methods. Studies not in English, dissertations/theses, reports or abstracts, letters to the editor and feature articles and articles intended as marketing or advertising material will be excluded. A publication timeframe of 1997-2017 will be applied. See Box 1 for the inclusion and exclusion criteria for the scoping review.

Box 1. Article inclusion and exclusion criteria for the scoping review

**Inclusion**
Types of publication: Peer-reviewed research articles. Full conference papers.<br>Language: English<br>Publication timeframe: 1997-2017<br>Types of research: Empirical studies using health-related data from social media, extracted or studied in situ, using both manual and automated methods.
**Exclusion**
Types of publication: Dissertations/theses; Reports or abstracts only; Letters to the editor; Marketing or advertising material; Reviews or editorials<br>Language: Not English<br>Types of research: Studies based on data from online sources other than social media (e.g. internet search histories, online news reports). Commercial research aimed at obtaining market intelligence or informing product promotion. Studies examining social media platforms, rather than using them as a source of data. Studies describing social media as a communication or broadcasting channel (e.g. for public health promotion).

Intermittent cross-checking by the second author will help to ensure the appropriate application of inclusion and exclusion criteria. In a further stage, the full-text of included articles will be independently assessed for eligibility by both authors and discrepancies resolved through discussion. Reasons for exclusion will be documented. These steps are described in Figure 2.

***[Figure 2.** Steps for identification, screening, eligibility and inclusion of studies in the scoping review]*

For peer review only

EndNote reference management software will be used to manage the records retrieved from searches. One reviewer will independently screen the generated citations with the help of EPPI-Reviewer 4 systematic review software, and undertake data extraction.

Stage 4: charting the data

The purpose of charting data in scoping reviews is to produce a descriptive summary of the results. For this stage, we have identified existing classification frameworks that correspond with the research questions listed previously. These will form the basis of our data charting form. The classification frameworks consider the purpose for which social media data are being used in the research, the method of data extraction (including any automated data mining tools used), the analytical-interpretive approach used (including stated theoretical perspectives), the locus of the research by academic institution and geographical scope of the data, the academic discipline associated with the research and whether/how ethical issues or guidelines are considered. Each of these existing classification frameworks and taxonomies are considered in further detail in Table 3 in relation to the specific research question. These classification frameworks are not intended to be totally prescriptive and additional emerging themes will be captured throughout the forthcoming analysis. These themes will later be used to identify gaps and inconsistencies represented in the existing frameworks, for future consideration and refinement.

Table 3. Existing classification frameworks that correspond with the research questions

Research question	Existing classification framework to be applied	How the classification framework will be applied
What year were the studies published?	Not specified	The year that the eligible study was published will be captured.
What terms are being used to describe the nature of the research?	Not specified	The terminology used to describe the aims or methodologies used in the study will be captured e.g. infoveillance, netnography, social listening.
Which academic communities are most active in conducting health research studies that use data from social media?	Scimago Journal Subject Areas [40] including 27 subject areas including medicine, computer science, health professions, business management and accounting as well as social sciences.	The journal in which eligible study is published, along with the first author's affiliation and source of funding for the study (if shown) will be classified according to the disciplinary taxonomy used by the Scimago Journal ranking portal. These categories are not mutually exclusive.
Where is the affiliation of the first author located?	World Bank Regional and Lending Groups for Countries includes seven regions and four lending groups [41]	The geographical location of the first author conducting the eligible study will be classified according to the regional and lending groups provided by the World Bank for 2017. These categories are not mutually exclusive.
What is the geographical scope of the sample of social media data analysed in the study?	World Bank Regional and Lending Groups for Countries includes seven regions and four lending groups [41]	The geographical location of the population sample analysed within the study will be classified according to the regional and lending groups provided by the World Bank for 2017. These categories are not mutually exclusive.
What is the purpose for which social media data are being used in the research?	Not specified	The purpose of the eligible study will be captured.
Which health topics are being studied?	The 19 health related topics which inform the World Health Organization's Sustainable Development Goals (SDG) [42]	The type of condition and health topic being studied in the eligible study will be classified according to the list of health related topics, which inform the WHO SDG. These categories are not mutually exclusive.
Which social media are used as a source of data?	Kaplan and Haenlein's eight types of social media [1]	The type of social media from which the data for the eligible study was sourced will be classified according to Kaplan and Haenlein's eight types of

For peer review only

Research question	Existing classification framework to be applied	How the classification framework will be applied
		social media. The name of the social media will also be captured.
How large are the studies and what is the unit of analysis applied?	Not specified	The sample size and unit of analysis of the eligible study will be captured.
How is the data from social media extracted and analysed and which proprietary tools were used?	Several types of analysis may be performed on social media data, ranging from simple descriptive statistics to qualitative research to automated real-time analytics at scale as described by Batrinca and Treleaven [43]. These types of analysis have been described in terms of computational techniques, such as natural language processing, and purposive approaches such as news analytics, opinion mining, data scraping, sentiment analysis and text analytics to name but a few.	Batrinca and Treleaven taxonomy of social media analysis techniques will be used to guide our classification of the broad approaches and specific techniques demonstrated in the eligible studies. This taxonomy may be refined as a result of new insights emerging during data extraction. We will also capture the named type of analysis performed in the eligible study as well as any reference to proprietary tools used.
How are ethical considerations applied in the published research?	Conway's taxonomy of ethics concepts for the use of Twitter in public health surveillance and research [44] and can be applied across all manner of social media.	The application of ethical concepts in the eligible studies will be classified according to the ten high level categories identified by Conway. These categories are not mutually exclusive.

Stage 5: collating, summarizing and reporting the results
The extracted data will be tabulated, with rows relating to articles, columns to classification variables and cells containing the relevant information. Both frequency analysis and trend analysis will be used to chart the classified results.

Frequency analysis
Using frequency analysis, the counts and percentages of eligible studies will be calculated. Studies will be grouped based on the classification frameworks and taxonomies applied. The result of this analysis will be a map of eligible studies represented in bubble plot, graph or tabular form.

Trend analysis
Trend analysis will be used to present the changing frequency of research over the past 20 years, based on the aforementioned classification criteria. A map showing the characteristics of included studies will be presented using both static and multi-media visualization tools, such as an animated bubble plot or graph.

Public and Patient Involvement
No patients or members of the public were involved in the protocol design or exploratory analyses, nor do we plan to include them in the conduct of the scoping review. Our results are nevertheless likely to be of interest to citizens who use social media and our decision to examine how researchers report ethical considerations reflects this concern. Dissemination will include accessible summaries and graphics, which we intend to make available to the public via social media.

Ethics and dissemination
Although some scoping reviews include a consultation phase, this one focuses on published research and online sources already in the public domain. No ethics approval is therefore required.
The completed scoping review will be submitted for publication to a peer-reviewed, interdisciplinary Open Access journal, in addition to conferences on public health and digital research. Findings will be presented using both static and multimedia visualisation tools.

Competing interests: The authors declare no competing interests.

Contributorship: JT designed the protocol and undertook the exploratory searches, with input from CP. The manuscript was drafted by JT and edited by CP.

For peer review only

Funding: JT is a self-funded PhD student at the University of Edinburgh, supervised by CP. She also works as a management consultant for Ernst and Young Switzerland although her employers are not involved in this research. CP is a collaborator on three RCUK-funded research programmes associated with this study: The Administrative Data Research Centre for Scotland, ESRC, Grant number ES/L007487/1; The Science and Practice of Social Machines EPSRC, EP/ J017728/1 and the Farr Institute for Health Informatics Research (Scotland) MRC, Grant number MR/K007017/1. The funders had no role in study design, data collection and analysis, decision to publish, or preparation of the manuscript.

Acknowledgements: Thanks to Marshall Dozier for advice on the search strategy and Santosh Vijaykumar for comments on a previous draft of the manuscript.

References

1. **Kaplan A, Haenlein M**: **Users of the world, unite! The challenges and opportunities of Social Media**. *Business Horizons* 2010, **53**(1):59-68.
2. **Complete History of Social Media: Then and Now** [https://smallbiztrends.com/2013/05/the-complete-history-of-social-media-infographic.html]
3. **Digital in 2017: Global Overview** [https://wearesocial.com/special-reports/digital-in-2017-global-overview]
4. **Social Media - Statistics and Facts** [https://www.statista.com/topics/1164/social-networks/]
5. **Current State of Social Media Research: From Practice to Theory #SMSociety15** [http://socialmedialab.ca/2015/current-state-of-social-media-research-from-practice-to-theory-part-1/]
6. **Social Media and Public Health Research** [https://bjerglund.files.wordpress.com/2012/11/final-social-media-and-public-health-research1.pdf]
7. Taylor J, Pagliari C: **Mining social media data: How are research sponsors and researchers addressing the ethical challenges?** *Research Ethics* 2017:1747016117738559.
8. Anstead N, O'Loughlin B: **Social Media Analysis and Public Opinion: The 2010 UK General Election**. *Journal of Computer-Mediated Communication* 2015, **20**(2):204-220.
9. **Social Media in Public Opinion Research: Report of the AAPOR Task Force on Emerging Technologies in Public Opinion Research** [https://www.aapor.org/AAPORKentico/AAPOR_Main/media/MainSiteFiles/AAPOR_Social_Media_Report_FNL.pdf]
10. Eysenbach G: **Infodemiology and Infoveillance: Framework for an Emerging Set of Public Health Informatics Methods to Analyze Search, Communication and Publication Behavior on the Internet**. *J Med Internet Res* 2009, **11**(1):e11.
11. Smith T: **The social media revolution**. *International Journal of Market Research* 2009, **51**(4):559.
12. Zafarani R, Ali Abbasi M, Liu H: **Social Media Mining: An Introduction**: Cambridge University Press; 2014.
13. **Social Analytics** [http://www.gartner.com/it-glossary/social-analytics]
14. Smart PR, Shadbolt NR: **Social Machines**. In: *Encyclopedia of Information Science and Technology.* edn. Edited by Khosrow-Pour M: IGI Global; 2014.
15. Kozinets RV: **Netnography**. In: *The International Encyclopedia of Digital Communication and Society.* edn.: John Wiley & Sons, Inc.; 2015.
16. Velasco E, Agheneza T, Denecke K, Kirchner G, Eckmanns TIM: **Social Media and Internet-Based Data in Global Systems for Public Health Surveillance: A Systematic Review**. *Milbank Quarterly* 2014, **92**(1):7-33.
17. Bernardo TM, Rajic A, Young I, Robiadek K, Pham MT, Funk JA: **Scoping review on search queries and social media for disease surveillance: a chronology of innovation**. *Journal of medical Internet research* 2013, **15**(7):e147.
18. Fung IC, Duke CH, Finch KC, Snook KR, Tseng PL, Hernandez AC, Gambhir M, Fu KW, Tse ZT: **Ebola virus disease and social media: A systematic review**. *American journal of infection control* 2016, **44**(12):1660-1671.
19. O'Shea J: **Digital disease detection: A systematic review of event-based internet biosurveillance systems**. *International journal of medical informatics* 2017, **101**:15-22.
20. Yan SJ, Chughtai AA, Macintyre CR: **Utility and potential of rapid epidemic intelligence from internet-based sources**. *International journal of infectious diseases : IJID : official publication of the International Society for Infectious Diseases* 2017, **63**:77-87.

For peer review only

21. Al-garadi MA, Khan MS, Varathan KD, Mujtaba G, Al-Kabsi AM: **Using online social networks to track a pandemic: A systematic review**. *Journal of Biomedical Informatics* 2016, **62**:1-11.
22. **A systematic literature review on event-based public health surveillance systems** [http://edoc.sub.uni-hamburg.de/haw/volltexte/2012/1614/pdf/lsab12_27.pdf]
23. Guy S, Ratzki-Leewing A, Bahati R, Gwadry-Sridhar F: **Social Media: A Systematic Review to Understand the Evidence and Application in Infodemiology**. In: *Electronic Healthcare: 2012// 2012; Berlin, Heidelberg*: Springer Berlin Heidelberg; 2012: 1-8.
24. Verhoef LM, Van de Belt TH, Engelen LJLPG, Schoonhoven L, Kool RB: **Social media and rating sites as tools to understanding quality of care: a scoping review**. *Journal of medical Internet research* 2014, **16**(2):e56.
25. Emmert M, Sander U, Pisch F: **Eight Questions About Physician-Rating Websites: A Systematic Review**. *J Med Internet Res* 2013, **15**(2):e24.
26. Golder S, Norman G, Loke YK: **Systematic review on the prevalence, frequency and comparative value of adverse events data in social media**. *British journal of clinical pharmacology* 2015, **80**(4):878-888.
27. Kazemi DM, Borsari B, Levine MJ, Dooley B: **Systematic review of surveillance by social media platforms for illicit drug use**. *Journal of public health (Oxford, England)* 2017:1-14.
28. Tursunbayeva A, Franco M, Pagliari C: **Use of social media for e-Government in the public health sector: A systematic review of published studies**. *Government Information Quarterly* 2017, **34**(2):270-282.
29. Patel R, Chang T, Greysen SR, Chopra V: **Social Media Use in Chronic Disease: A Systematic Review and Novel Taxonomy**. *The American Journal of Medicine*, **128**(12):1335-1350.
30. Golder S, Ahmed S, Norman G, Booth A: **Attitudes Toward the Ethics of Research Using Social Media: A Systematic Review**. *Journal of medical Internet research* 2017, **19**(6):e195.
31. Arksey H, O'Malley L: **Scoping studies: towards a methodological framework**. *International Journal of Social Research Methodology* 2005, **8**(1):19-32.
32. Levac D, Colquhoun H, O'Brien KK: **Scoping studies: advancing the methodology**. *Implementation Science : IS* 2010, **5**:69-69.
33. **Chapter 11: Scoping Reviews.** [Available from https://reviewersmanual.joannabriggs.org/]
34. Armstrong R, Hall Bj Fau - Doyle J, Doyle J Fau - Waters E, Waters E: **Cochrane Update. 'Scoping the scope' of a cochrane review**. (1741-3850 (Electronic)).
35. Horsley T, Dingwall O, Sampson M: **Checking reference lists to find additional studies for systematic reviews**. *Cochrane Database of Systematic Reviews* 2011(8).
36. **Search Engine Market Share** [https://netmarketshare.com/search-engine-market-share.aspx?options=%7B%22filter%22%3A%7B%22%24and%22%3A%5B%7B%22deviceType%22%3A%7B%22%24in%22%3A%5B%22Desktop%2Flaptop%22%5D%7D%7D%5D%7D%2C%22dateLabel%22%3A%22Trend%22%2C%22attributes%22%3A%22share%22%2C%22group%22%3A%22searchEngine%22%2C%22sort%22%3A%7B%22share%22%3A-1%7D%2C%22id%22%3A%22searchEnginesDesktop%22%2C%22dateInterval%22%3A%22Monthly%22%2C%22dateStart%22%3A%222017-03%22%2C%22dateEnd%22%3A%222018-02%22%2C%22segments%22%3A%22-1000%22%7D]
37. Lam MT, McDiarmid M: **Increasing number of databases searched in systematic reviews and meta-analyses between 1994 and 2014**. *Journal of the Medical Library Association : JMLA* 2016, **104**(4):284-289.
38. **List of social networking websites** [http://en.wikipedia.org/wiki/List_of_social_networking_websites]
39. **Top 25 Health and Medical Social Media Sites** [http://mastersinhospitaladministration.com/2011/top-25-health-and-medical-social-media-sites/]
40. **Scimago Journal & Country Rank** [http://www.scimagojr.com/journalrank.php]
41. **World Bank Country and Lending Groups** [https://datahelpdesk.worldbank.org/knowledgebase/articles/906519-world-bank-country-and-lending-groups]
42. **World Health Statistics 2016 data visualizations dashboard: Monitoring health for SDGs** [http://apps.who.int/gho/data/node.sdg.tp-1?lang=en]
43. Batrinca B, Treleaven PC: **Social media analytics: a survey of techniques, tools and platforms**. *AI & SOCIETY* 2015, **30**(1):89-116.

For peer review only

44. Conway M: **Ethical Issues in Using Twitter for Public Health Surveillance and Research: Developing a Taxonomy of Ethical Concepts From the Research Literature**. *J Med Internet Res* 2014, **16**(12):e290.

For peer review only

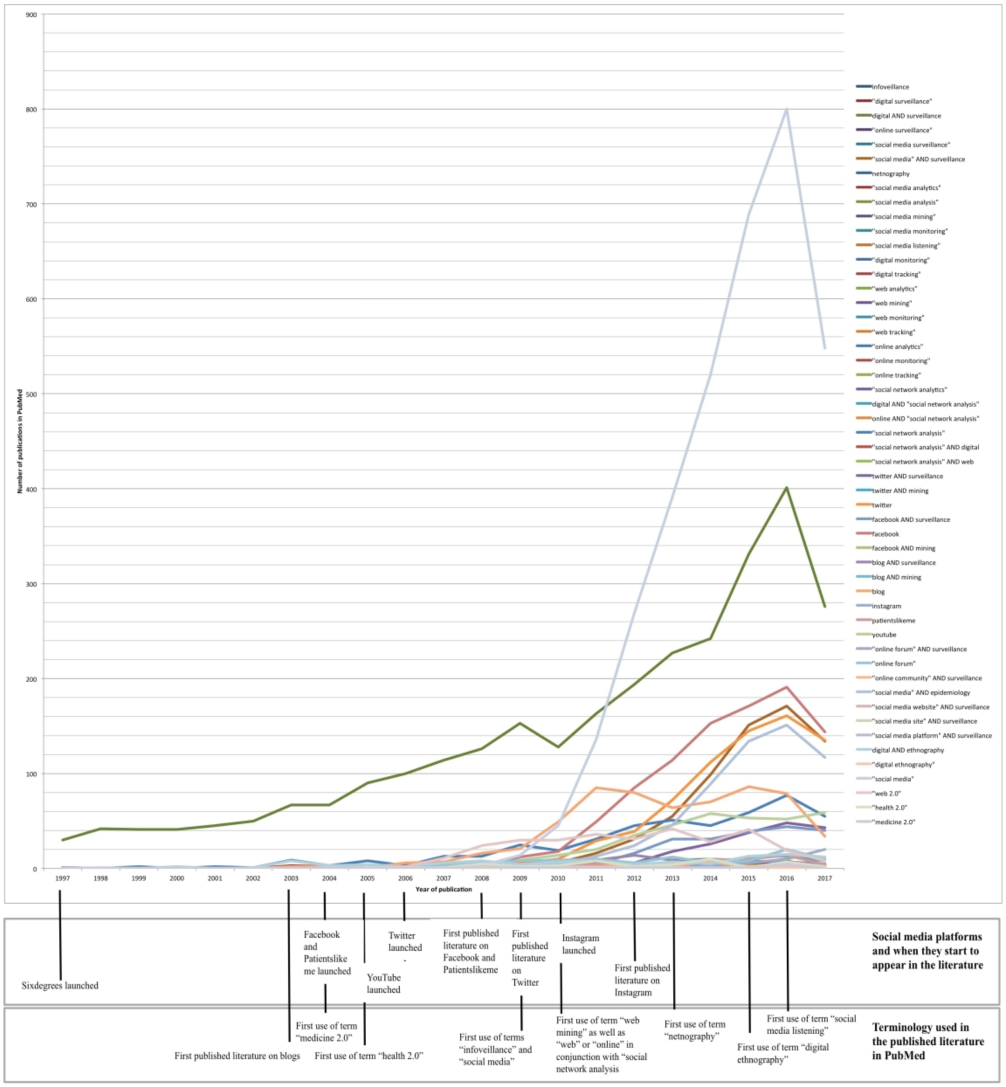

Figure 1. Results of the search queries by year

245x265mm (300 x 300 DPI)

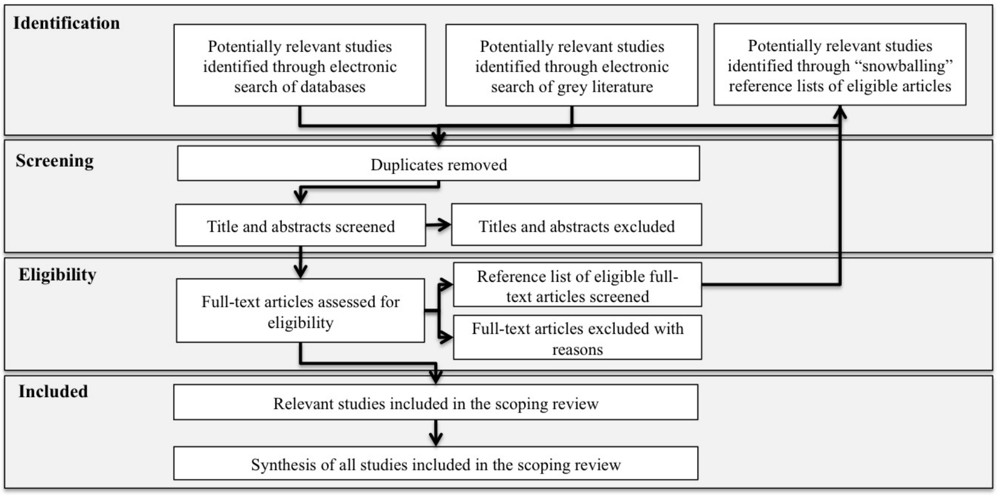

Figure 2. Steps for identification, screening, eligibility and inclusion of studies in the scoping review

113x56mm (300 x 300 DPI)

Appendix 1. Scoping and systematic reviews on the secondary use of data from social media

	Systematic or Quasi-Systematic Scoping Review	Review Aim/Objective/Main Focus	Number of Databases Used	Key Findings
1	Velasco, E., Agheneza, T., Denecke, K., Kirchner, G., Eckmanns, T. Social media and internet-based data in global systems for public health surveillance: a systematic review. Milbank Q. 2014; 92(1): 7-33	To explore how useful are data from social media and the Internet, and what is the potential to enhance surveillance.	3	• No event-based surveillance systems currently used in national surveillance programs<br>• Existing systems have limitations<br>• There is a need for further development of automated technologies that monitor health-related information on the Internet<br>• The dissemination to health authorities of new information about health events is not always efficient and could be improved.<br>• No comprehensive evaluations show whether event-based surveillance systems have been integrated into actual epidemiological work during real-time health events.
2	Golder, S., Norman, G., Loke, Y.K. Systematic review on the prevalence, frequency and comparative value of adverse events data in social media British Journal of Clinical Pharmacology. 2015; 80(4); 878-88	To summarize the prevalence, frequency and comparative value of information on the adverse events of healthcare interventions from user comments and videos in social media.	16 databases and 2 internet search engines	• The prevalence of adverse events reports on social media varies from 0.2% to 8% of posts.<br>• 'Mild' and symptom-related adverse events are over-represented in social media and laboratory test abnormalities and 'serious' adverse events are under- represented compared with other data sources.<br>• The question as to whether searching social media for adverse events data is a valuable use of resources, resulting in improved patient outcomes remains unanswered.
3	Verhoef, L.M., Van de Belt, T.H., Engelen, L.J.L.P.G., Schoonhoven, L., Kool, R.B. Social media and rating sites as tools to understanding quality of care: a scoping review J Med Internet Res. 2014; 16(2); e56	To explore the relationship between data from social media and quality of care.	4	• There is a relationship between information on social media and quality of health care<br>• Drawbacks exist, since rating is anonymous and vulnerable to fraud. They are often based on only a few reviews and are predominantly positive.<br>• People providing feedback on health care via social media are presumably not always representative for the patient population.
4	Bernardo, T.M., Rajic, A., Young, I., Robiadek, K., Pham, M.T., Funk, J.A. Scoping review on search queries and social media for disease surveillance: a chronology of innovation J Med Internet Res. 2013; 15(7); e147	To assess the current state of knowledge regarding the use of search queries and social media for disease surveillance in order to inform future work on early detection and more effective mitigation of the effects of foodborne illness.	1 database and 2 internet search engines	• The use of search queries and social media for disease surveillance are relatively recent phenomena (first reported in 2006).<br>• Both the tools themselves and the methodologies for exploiting them are evolving over time.<br>• While their accuracy, speed, and cost compare favorably with existing surveillance systems, the primary challenge is to refine the data signal by reducing surrounding noise.
5	Fung, Tse, Cheung, Miu, & Fu. (2014). Ebola and the social media. The Lancet,	To systematically reviewed existing research pertinent to Ebola virus disease and social media, especially	6	• Included 7 articles on Twitter, 1 on Facebook, 3 on YouTube, 1 on Instagram & Flickr.

For peer review only

	Systematic or Quasi-Systematic Scoping Review	Review Aim/Objective/Main Focus	Number of Databases Used	Key Findings
	384(9961), 2207.	to identify the research questions and the methods used to collect and analyze social media.		• 11 articles examined Ebola posts' content themes, meta-data or user characteristics.<br>• One article examined how Ebola-related news videos influenced Twitter traffic.
6	Golder S, Ahmed S, Norman G, Booth A Attitudes Toward the Ethics of Research Using Social Media: A Systematic Review J Med Internet Res 2017;19(6):e195	To ascertain attitudes on the ethical considerations of using social media as a data source for research as expressed by social media users and researchers.	16 data bases and 2 internet search engines	• Attitudes varied from overly positive with people expressing the views about the essential nature of such research for the public good, to very concerned with views that social media research should not happen.<br>• Underlying reasons for this variation related to issues such as the purpose and quality of the research, the researcher affiliation, and the potential harms.<br>• The methods used to conduct the research were also important.<br>• Many respondents were positive about social media research while adding caveats such as the need for informed consent or use restricted to public platforms only.
7	Kazemi, D., Borsari, B., Levine, M., & Dooley, B. (2017). Systematic review of surveillance by social media platforms for illicit drug use. Journal of Public Health (Oxford, England), 1-14.	To review the ability of social media to better recognize illicit drug use trends was addressed.	9	Selected studies used a range of social media tools/applications, including message boards, Twitter and blog/forums/platform discussions.
8	O'Shea, Jesse. (2017). Digital disease detection: A systematic review of event-based internet biosurveillance systems. International Journal of Medical Informatics, 101, 15-22.	To update the current state of knowledge on event-based Internet biosurveillance systems by identifying all systems, including current functionality, with hopes to aid decision makers with whether to incorporate new methods into comprehensive programmes of surveillance.	3	• Each system uses different innovative technology and data sources to gather data, process, and disseminate data to detect infectious disease outbreaks.
9	Yan, Chughtai, & Macintyre. (2017). Utility and potential of rapid epidemic intelligence from internet-based sources. International Journal of Infectious Diseases, 63, 77-87.	To identify and summarise the types of Internet-based surveillance methods studied in recent literature. A secondary aim was to identify and summarise the timeliness and accuracy outcomes of Internet-based methods described in literature.	5	• Studies used search queries, social media posts and approaches derived from existing Internet-based systems for early epidemic alerts and real-time monitoring.<br>• Most studies noted improved timeliness compared to official reporting<br>• Internet-based methods showed variable correlation strength with official datasets, with some methods showing reasonable accuracy.
10	Al-Garadi, Khan, Varathan, Mujtaba, & Al-Kabsi. (2016). Using online social networks to track a pandemic: A systematic review. Journal of Biomedical Informatics, 62, 1-11.	To conduct a systematic review of the use of online social networks to track a pandemic.	5	• Identified the extent of using online social networks to track a pandemic.<br>• Online social networks contain significant information to track a pandemic almost in real time.<br>• There are challenges and future implications.
11	Agheneza T. A Systematic Review of Event-Based Public Health Surveillance Systems. Hamburg: Faculty	To identify all the event-based surveillance systems that exist and where they are based.	3	• 13 event-based systems were identified, and with 10 of the 13 systems evaluated.<br>• N. America is the leading

For peer review only

	Systematic or Quasi-Systematic Scoping Review	Review Aim/Objective/Main Focus	Number of Databases Used	Key Findings
	of Life Sciences, Hamburg University of Applied Sciences; 2011.			continent with about 77 % of the event-based systems followed by Europe and lastly Asia. No system was found in Africa, Australia and S. America.
12	Emmert M, Sander U, Pisch F. Eight questions about physician-rating websites: a systematic review. J Med Internet Res. 2013;15(2):e24.	To identify frequently discussed issues about physician-rating websites.	7	• The current usage of physician-rating websites is still low but is increasing.<br>• International data show that 1 out of 6 physicians has been rated, and approximately 90% of all ratings on physician-rating websites were positive.<br>• Could not find any evidence of "doctor-bashing".<br>• Physicians should not ignore these websites, but rather, monitor the information available and use it for internal and external purposes.
13	Guy S, Ratzki-Leewing A, Bahati R, Gwadry-Sridhar F. Social media: A systematic review to understand the evidence and application in infodemiology. Lect Notes Inst Comput Sci Soc Informatics Telecommun Eng. 2012;91:1–8.	To systematically review the literature utilizing social media as a source for disease prediction and surveillance.	2	• Open-source micro-blogging sites can be used for influenza-like-illness monitoring.
14	Tursunbayeva, Franco, & Pagliari. (2017). Use of social media for e-Government in the public health sector: A systematic review of published studies. Government Information Quarterly, 34(2), 270-282.	To investigate the adoption and use of social media by public health organizations	4	• Evidence-base is mostly descriptive, unidisciplinary and lacks the theoretical depth seen in other branches of e-Government research.<br>• The lack of robust evidence makes it difficult to draw conclusions about the effectiveness of these approaches in the public health sector
15	Patel, Chang, Greysen, & Chopra. (2015). Social Media Use in Chronic Disease: A Systematic Review and Novel Taxonomy. The American Journal of Medicine, 128(12), 1335-1350.	To evaluate clinical outcomes from applications of contemporary social media in chronic disease; to develop a conceptual taxonomy to categorize, summarize, and then analyze the current evidence base; and to suggest a framework for future studies	1	• The overall impact of social media on chronic disease was variable.<br>• Among studies that showed benefit, most used either Facebook or blogs, and were based within the domain of support.

For peer review only

Appendix 2. Total number of publications by search query

Search query	Total number of publications from 1997-2017
"social media" AND (health OR illness OR disease) AND human	3425
digital AND surveillance AND (health OR illness OR disease) AND human	2935
facebook AND (health OR illness OR disease) AND human	939
twitter AND (health OR illness OR disease) AND human	709
"social media" AND surveillance AND (health OR illness OR disease) AND human	665
blog AND (health OR illness OR disease) AND human	602
"social media" AND epidemiology AND (disease OR health OR illness) AND human	581
"social network analysis" AND (disease OR health OR illness) AND human	462
youtube AND (health OR illness OR disease) AND human	351
"web 2.0" AND (health OR illness OR disease) AND human	312
facebook AND surveillance AND (health OR illness OR disease) AND human	206
twitter AND surveillance AND (health OR illness OR disease) AND human	187
digital AND ethnography AND (health OR illness OR disease) AND human	110
"online forum" AND (health OR illness OR disease) AND human	103
blog AND surveillance AND (health OR illness OR disease) AND human	76
patientslikeme AND (health OR illness OR disease) AND human	60
"online monitoring" AND (disease OR health OR illness) AND human	48
"health 2.0" AND (health OR illness OR disease) AND human	46
twitter AND mining AND (health OR illness OR disease) AND human	43
instagram AND (health OR illness OR disease) AND human	40
online AND "social network analysis" AND (disease OR health OR illness) AND human	29
"online forum" AND surveillance AND (health OR illness OR disease) AND human	25
"online community" AND surveillance AND (health OR illness OR disease) AND human	25
infoveillance AND (disease OR health OR illness) AND human	24
"online surveillance" AND (disease OR health OR illness) AND human	23
"social network analysis" AND web AND (disease OR health OR illness) AND human	23
"digital surveillance" AND (disease OR health OR illness) AND human	16
netnography AND (disease OR health OR illness) AND human	14
"medicine 2.0" AND (health OR illness OR disease) AND human	14
"social media analysis" AND (disease OR health OR illness) AND human	12
"online tracking" AND (disease OR health OR illness) AND human	12
"web analytics" AND (disease OR health OR illness) AND human	10
"social media analytics" AND (disease OR health OR illness) AND human	9
"social media monitoring" AND (disease OR health OR illness) AND human	9
blog AND mining AND (health OR illness OR disease) AND human	9
"web mining" AND (disease OR health OR illness) AND human	8
"digital monitoring" AND (disease OR health OR illness) AND human	7
facebook AND mining AND (health OR illness OR disease) AND human	7
"social media mining" AND (disease OR health OR illness) AND human	6
"web monitoring" AND (disease OR health OR illness) AND human	6

For peer review only

Search query	Total number of publications from 1997-2017
"social media platform" AND surveillance AND (health OR illness OR disease) AND human	6
"social media surveillance" AND (disease OR health OR illness) AND human	4
"web tracking" AND (disease OR health OR illness) AND human	4
digital AND "social network analysis" AND (disease OR health OR illness) AND human	4
"social network analysis" AND digital AND (disease OR health OR illness) AND human	4
"digital ethnography" AND (health OR illness OR disease) AND human	4
"digital tracking" AND (disease OR health OR illness) AND human	3
"online analytics" AND (disease OR health OR illness) AND human	3
"social media site" AND surveillance AND (health OR illness OR disease) AND human	3
"social media listening" AND (disease OR health OR illness) AND human	2
"social network analytics" AND (disease OR health OR illness) AND human	2
"social media website" AND surveillance AND (health OR illness OR disease) AND human	2
"web surveillance" AND (disease OR health OR illness) AND human	0
"social network surveillance" AND (disease OR health OR illness) AND human	0
"social media observing" AND (disease OR health OR illness) AND human	0
"social media tracking" AND (disease OR health OR illness) AND human	0
"digital observing" AND (disease OR health OR illness) AND human	0
"digital analytics" AND (disease OR health OR illness) AND human	0
"digital mining" AND (disease OR health OR illness) AND human	0
"digital listening" AND (disease OR health OR illness) AND human	0
"web observing" AND (disease OR health OR illness) AND human	0
"web listening" AND (disease OR health OR illness) AND human	0
"online observing" AND (disease OR health OR illness) AND human	0
"online mining" AND (disease OR health OR illness) AND human	0
"online listening" AND (disease OR health OR illness) AND human	0
"social network observing" AND (disease OR health OR illness) AND human	0
"social network mining" AND (disease OR health OR illness) AND human	0
"social network monitoring" AND (disease OR health OR illness) AND human	0
"social network listening" AND (disease OR health OR illness) AND human	0
"social network tracking" AND (disease OR health OR illness) AND human	0
linkedin AND (health OR illness OR disease) AND human	0
"sixdegrees" AND (health OR illness OR disease) AND human	0

For peer review only

BMJ Open

A Comprehensive Scoping Review of Health Research Using Social Media Data (Protocol)

Journal:	*BMJ Open*
Manuscript ID	bmjopen-2018-022931.R2
Article Type:	Protocol
Date Submitted by the Author:	29-Jul-2018
Complete List of Authors:	Taylor, Joanna; University of Edinburgh, eHealth Research Group, Usher Institute of Population Health Sciences and Informatics; Ernst and Young<br>Pagliari, Claudia; University of Edinburgh, eHealth Research Group, Usher Institute for Population Health Sciences
**Primary Subject Heading**:	Health informatics
Secondary Subject Heading:	Public health
Keywords:	Social Media, eHealth, Digital Research, Public Health Informatics, Data Science, Digital Health

SCHOLARONE™
Manuscripts

A Comprehensive Scoping Review of Health Research Using Social Media Data (Protocol)

Joanna Taylor and Claudia Pagliari

Corresponding Author: Claudia Pagliari, eHealth Research Group, Usher Institute for Population Health Sciences and Informatics, University of Edinburgh, Medical School, Teviot Place, Edinburgh EH8 9AG, UK.
Email: claudia.pagliari@ed.ac.uk
Tel: 0131 6509464
Fax: 0131 6509119

Joanna Taylor
Usher Institute for Population Health Sciences and Informatics, The University of Edinburgh, UK
Ernst and Young Ltd, Switzerland

Claudia Pagliari
Usher Institute for Population Health Sciences and Informatics, The University of Edinburgh, UK
claudia.pagliari@ed.ac.uk

Keywords: Social Media [MeSH]**, eHealth, Digital Research, Public Health Informatics** [MeSH]**, Data Science**

For peer review only

Abstract

Introduction: The rising popularity of social media, since their inception around 20 years ago, has been echoed in the growth of health-related research using data derived from them. This has created a demand for literature reviews to synthesise this emerging evidence-base and inform future activities. Existing reviews tend to be narrow in scope, with limited consideration of the different types of data, analytical methods and ethical issues involved. There has also been a tendency for research to be siloed within different academic communities (e.g. computer science, public health), hindering knowledge translation. To address these limitations, we will undertake a comprehensive scoping review, to systematically capture the broad corpus of published, health-related research based on social media data. Here we present the review protocol and the pilot analyses used to inform it.

Methods: A version of Arksey and O'Malley's five-stage scoping review framework will be followed: (1) identifying the research question; (2) identifying the relevant literature; (3) selecting the studies; (4) charting the data; and (5) collating, summarizing and reporting the results. To inform the search strategy we developed an inclusive list of keyword combinations related to social media, health and relevant methodologies. The frequency and variability of terms were charted over time and cross referenced with significant events, such as the advent of Twitter. Five leading health, informatics, business and cross-disciplinary databases will be searched: PubMed, Scopus, ACM, IEEE and Applied Social Sciences Index and Abstracts, alongside the Google search engine. There will be no restriction by date.

Ethics and dissemination: The review focuses on published research in the public domain therefore no ethics approval is required. The completed review will be submitted for publication to a peer-reviewed, interdisciplinary Open Access journal, and conferences on public health and digital research.

Article Summary

Strengths and limitations of this study

- The proposed scoping review addresses the need for a comprehensive, cross-disciplinary synthesis of health-related research using social media data.
- A 5-stage scoping review protocol was developed, which was informed by an exploratory analysis of existing reviews, terminologies and published taxonomies.
- This revealed the changing usage of relevant terms over time, the types of keyword combinations most likely to yield eligible studies, and the time-lag between the launch of new social media platforms and published research using data derived from them.
- The findings of the scoping review will be communicated using both static and multi-media visualisation tools.

For peer review only

Introduction - Context and coverage of the existing review literature

Social media are online, often mobile, platforms that support the creation and exchange of user-generated content [1]. They include generic platforms for networking, information sharing and collaboration (e.g. Facebook, Twitter, YouTube, Linkedin) and online forums aimed at specific communities (e.g. Patientslikeme, Mumsnet). The first modern day social media platform, sixdegrees, was launched in 1997 and allowed users to connect with friends and family through sending messages and posts on bulletin boards [2]. Since then the use of social media has become increasingly common [3] with an estimated 2.5 billion of the global population estimated to be using them as of 2017 [4].

As social media have become an integral part of people's lives, research focusing on social media has also evolved and is taking place in a number of domains. A bibliometric content analysis of key words that appeared in the abstracts of 14,500 journal articles and conference papers identified five main areas in which a high volume of research already exists. This includes research related to health, educational uses of social media, computing and computer science methods, business, organizational and marketing topics, and political and social engagement [5].

Uses of social media in the context of research can be described according to the broad categories identified by Bjerglund-Andersen and Söderqvist (dissemination, discussion/networking, public engagement, teaching, research/data collection) [6]. Taylor and Pagliari (2017) [7] recently separated the last of these into social media as a *source of data* for research and as a tool for *the conduct of research*. These 'secondary uses' of social media data include analyses of trends, associations and sentiments in users' postings, as well as interactions and networks [8, 9].

This new source of 'big data' has triggered scientific developments in a number of areas, including health. The term "infoveillance" was coined by Eysenbach in 2009 to describe the automated harvesting and analysis of internet searches and social media postings as an alternative approach for health and disease surveillance [10]. Other terms have since been introduced across academia and industry to describe the use of such data for gleaning insights about behavioural trends, determining the impact of interventions or predicting future events. These include "social media listening" [11], "social media mining" [12], "social analytics" [13], "social machines" [14] and "netnography" [15], to name but a few.

Based on an iterative search using PubMed we identified an existing corpus of 15 systematic, quasi-systematic and scoping reviews on the secondary use of social media data for health research, examples of which are summarized in Table 1 [see Appendix 1 for the full list, summarised according to their aims, number of databases and findings]. Amongst these are reviews on approaches to health surveillance [16-23] quality of healthcare services [24, 25], adverse event monitoring [26], illicit drug use [27], eGovernment [28], chronic conditions [29] and ethics [30].

Table 1. Existing systematic, quasi-systematic or scoping reviews indexed in PubMed.

Topic	Examples of Systematic, Quasi-systematic or Scoping Reviews
Disease surveillance	Social media and internet-based data in global systems for public health surveillance: a systematic review [16]
	Scoping review on search queries and social media for disease surveillance: a chronology of innovation [17]
	Ebola and the social media. [18]
	Digital disease detection: A systematic review of event-based internet biosurveillance systems. [19]
	Utility and potential of rapid epidemic intelligence from internet-based sources. [20]
	Using online social networks to track a pandemic: A systematic review. [21]
	A Systematic Review of Event-Based Public Health Surveillance Systems. [22]
	Social media: A systematic review to understand the evidence and application in infodemiology. [23]
Adverse event monitoring	Systematic review on the prevalence, frequency and comparative value of adverse events data in social media [26]
Quality of healthcare services	Social media and rating sites as tools to understanding quality of care: a scoping review [24]
	Eight questions about physician-rating websites: a systematic review. [25]
Illicit drug use	Systematic review of surveillance by social media platforms for illicit drug use. [27]
eGovernment	Use of social media for e-Government in the public health sector: A systematic review of published studies. [28]
Chronic disease	Social Media Use in Chronic Disease: A Systematic Review and Novel Taxonomy [29]
Ethics	Attitudes Toward the Ethics of Research Using Social Media: A Systematic Review [30]

For peer review only - http://bmjopen.bmj.com/site/about/guidelines.xhtml

A 2013 scoping review by Bernardo et al. on the use of internet search queries and social media for disease surveillance identified the first study as has having been published in 2006 and described how techniques for exploiting this data are evolving to increase the accuracy of signal detection [17]. Although insightful in understanding the chronology of this type of innovation, the review searched only 1 database and focused on the surveillance of foodborne illness. A 2014 systematic review by Velasco et al. on the impact and use of data from social media for public health surveillance went further, concluding that existing surveillance systems are limited and there is a need for automated technologies to monitor health related information on the Internet, although it did not specify which types of systems were analysed [16]. O'Shea et al.'s 2017 systematic review of event-based internet biosurveillance systems describes the wide variety of technologies and data sources for gathering, processing and disseminating data to detect infectious disease outbreaks [19]. These reviews, alongside the others listed, focus predominantly on infectious disease. A systematic review on social media for chronic disease exists [29] which focuses on understanding the clinical outcomes associated with using such technologies for patient support, education and disease management across different conditions. However this is limited by the inclusion of only database, and did not examine the type of methods or tools used to extract and analyse social media data, the academic discipline and setting of the research or the ethical issues considered. With respect to the latter, a recent systematic review by Golder et al., analysed studies reporting attitudes to research using social media data. This revealed wide variation in attitudes, from the very positive to the very concerned, depending on the purpose and quality of the research, researcher affiliation, the potential for harm and the methods used [30]. Although it used an impressive 16 databases, this review did not examine regional, disciplinary, condition or health topic specific variations and the authors note that the demographic characteristics of respondents was unclear in most studies. As noted in a recent review of UK research ethics guidelines and published health research, growing public awareness of the misuse of social media for marketing and algorithmic prediction, are forcing policymakers to look more closely at this issue [7].

In light of these gaps, we set out to undertake a comprehensive scoping review aimed at capturing and profiling a broad corpus of published multi-disciplinary research in which data obtained from social media have been used to monitor, understand or evaluate aspects of health and disease. In this paper, we describe the formal protocol for the comprehensive scoping review, alongside the preliminary analyses undertaken to inform each stage.

Methods and analysis

Scoping reviews are a type of quasi-systematic review that are increasingly used for understanding research on emerging innovations, which may be poorly indexed, distributed across published and grey literature or located in different academic disciplines [31-33]. They typically progress in five key stages (1) identifying the research question; (2) identifying the relevant studies; (3) selecting the studies; (4) charting the data; and (5) collating, summarizing and reporting the results. Developing an *a priori* review protocol can be useful for managing this complexity, while formative research can aid the design of such protocols by identifying relevant terminologies, topics and evidence sources. Scoping reviews are mainly aimed at mapping the evidence landscape rather than establishing the effectiveness of particular interventions and typically do not involve critical appraisal of study methodology or detailed extraction of outcomes data [34].

Stage 1: identifying the research question

In addition to the overarching review objective articulated in the introduction, several specific questions will be used to guide our analysis of existing research evidence, as listed in Table 2.

Table 2. Research Questions

Aspect	List of questions
General	What is the total number of studies published by year?
	What terms are being used to describe the nature of this research?
	Which academic communities are most active in health research using data from social media?
	Where are study authors located, according to their affiliation?
	What is the geographical scope of the social media data analysed in these studies?
	For what purposes are social media data being used in this research?
Topic	Which health topics are being studied?
SM Type	Which social media platforms or sites are being used as sources of data?
Extract & analysis	What units of analysis are being applied?
	How are data from social media being extracted and analysed and which proprietary tools are being used?
Ethics	How are ethical considerations applied in the published research?

For peer review only

Stage 2: identifying relevant literature

Comprehensive scoping reviews aim to capture literature from a range of electronic databases, reference lists, and grey literature. As such, our approach will include:

- A systematic search of peer-reviewed studies using five health, informatics, business and cross-disciplinary electronic databases: PubMed, Scopus, Association of Computer Machinery (ACM), Institute of Electrical and Electronics Engineers (IEEE) and Applied Social Sciences Index and Abstracts (ASSIA).
- 'Snowballing' from article reference lists [35] will be used to identify additional studies that may have not been indexed in the online research databases.
- Searching grey literature from the Internet using the most widely used search engine, Google [36]. The first 20 Google results yielded by each search string will be reviewed, as this search engine displays the results by relevance.

The search strategy for each of the databases was defined in consultation with a senior librarian. Our five electronic databases and one internet search engine takes account of time and funding constraints, although the sources targeted are likely to capture most of the relevant literature [37].

Formative searches:

Given the breadth and changing popularity of terms related to social media and social media mining, we undertook a formative analysis to understand those most likely to yield articles relevant to our review objectives. We first created an extensive list based on the search strings specified in a recent systematic review of social media in the context of e-government in public health [28], supplemented with terms captured from iterative online searches. The list included generic terms related to social media (e.g. digital, blog, social network, Web 2.0), named social media (e.g. Twitter, Facebook), types of social media mining (e.g. surveillance, scraping, listening, infoveillance) and analytics (e.g. netnography, social network analysis) [see Appendix 2 for the full list]. We then ran separate search queries for each term in PubMed, constructed as [*term*] *AND (health OR illness OR disease) AND human*. Searches yielding one or more hits were charted over time, to examine their changing frequency in the research literature. Out of the 72 terms tested in PubMed, 52 produced hits. The timeline was cross-referenced with key historical events, such as the introduction of new platforms or new methodologies. The results are shown in Figure 1.

*[**Figure 1.** Results of the search queries by year]*

As can be seen from Figure 1, 91% of relevant studies were published in the past 9 years (11,154 results between 2009-2017, vs. 1,048 between 1997-2008). Not surprisingly, the search term "social media" produced the highest number of results overall compared to other search queries. Regarding specific platforms, searches with sixdegrees and Linkedin produced no results, compared with other social media such as Facebook (1152 hits, between 2008-2017), Twitter (939 hits, between 2009-2017), YouTube (351 hits, between 2008-2017), Patientslikeme (60 hits, between 2008-2017), and Instagram (40 hits, between 2012-2017). We also observed a time lag of four years between when Facebook, Patientslikeme and Twitter were launched in 2004, 2004 and 2006 respectively and the first research article was published, while the equivalent latency for YouTube and Instagram was two years. The individual search terms "surveillance" (665), "epidemiology" (581) and "ethnography" (110) produced the highest yields, compared with terms specific to digital research methods. With respect to the latter, temporal changes in the use of terms such as "infoveillance", "netnography", "social media listening", "social media analytics" and "social media mining" indicate the evolution of innovations and research perspectives, however including such specialist terms was not critical for identifying relevant studies.

As previously noted, a systematic search of five health, informatics, business and cross-disciplinary electronic databases - PubMed, Scopus, ACM, IEEE and ASSIA – will be undertaken to identify relevant peer-reviewed studies. Below is the search query that will be used to interrogate these sources. This takes account of insights gathered during our formative analyses:

- Named social media [38], including health specific social media sites [39]. All searches that do not yield results will be excluded
- OR "web 2.0" OR "social media" OR "blog" OR "microblog" OR "wiki" OR "virtual world" OR "discussion forum" OR "online community"
- AND "surveillance" OR "epidemiology" OR "infoveillance" OR "ethnography" OR "netnography" OR "mining"

For peer review only

- AND (“health” OR “disease” OR “illness”)
- NOT “animal”

For Google searches the advanced search option will be used with English selected as the preferred language. All regions will be included and the following search strings will be used:

- “social media, surveillance, health, NOT animal”
- “social media, surveillance, disease, NOT animal”
- “social media, data mining, health, NOT animal”
- “social media, data mining, disease, NOT animal”
- “social media, epidemiology, health, NOT animal”
- “social media, epidemiology, disease, NOT animal”

Stage 3: study selection
One researcher will conduct the systematic search of electronic databases and grey literature. Studies will be selected after the abstracts and titles, identified via the electronic searches and “snowballing” technique, have been independently screened for relevance by two researchers based on the specified inclusion criteria.

Inclusion and exclusion criteria
Peer-reviewed journal articles and conference papers will be included where these describe empirical research using data from social media such as Twitter or Facebook, whether extracted or studied situ, using either manual or automated methods. Studies not in English, dissertations/theses, reports or abstracts, letters to the editor and feature articles and articles intended as marketing or advertising material will be excluded. A publication timeframe of 1997-2017 will be applied. See Box 1 for the inclusion and exclusion criteria for the scoping review.

Box 1. Article inclusion and exclusion criteria for the scoping review

**Inclusion**
Types of publication: Peer-reviewed research articles. Full conference papers.<br>Language: English<br>Publication timeframe: 1997-2017<br>Types of research: Empirical studies using health-related data from social media, extracted or studied in situ, using both manual and automated methods.
**Exclusion**
Types of publication: Dissertations/theses; Reports or abstracts only; Letters to the editor; Marketing or advertising material; Reviews or editorials<br>Language: Not English<br>Types of research: Studies based on data from online sources other than social media (e.g. internet search histories, online news reports). Commercial research aimed at obtaining market intelligence or informing product promotion. Studies examining social media platforms, rather than using them as a source of data. Studies describing social media as a communication or broadcasting channel (e.g. for public health promotion).

Intermittent cross-checking by the second author will help to ensure the appropriate application of inclusion and exclusion criteria. In a further stage, the full-text of included articles will be independently assessed for eligibility by both authors and discrepancies resolved through discussion. Reasons for exclusion will be documented. These steps are described in Figure 2.

***[Figure 2.** Steps for identification, screening, eligibility and inclusion of studies in the scoping review]*

EndNote reference management software will be used to manage the records retrieved from searches. One reviewer will independently screen the generated citations with the help of EPPI-Reviewer 4 systematic review software, and undertake data extraction.

Stage 4: charting the data
The purpose of charting data in scoping reviews is to produce a descriptive summary of the results. For this stage, we have identified existing classification frameworks that correspond with the research questions listed previously. These will form the basis of our data charting form. The classification frameworks consider the purpose for which social media data are being used in the research, the method of data extraction (including any automated data mining tools used), the analytical-interpretive approach used (including stated theoretical perspectives), the locus of the research by academic institution and geographical scope of the data, the academic discipline associated with the research and whether/how

For peer review only

ethical issues or guidelines are considered. Each of these existing classification frameworks and taxonomies are considered in further detail in Table 3 in relation to the specific research question. These classification frameworks are not intended to be totally prescriptive and additional emerging themes will be captured throughout the forthcoming analysis. These themes will later be used to identify gaps and inconsistencies represented in the existing frameworks, for future consideration and refinement.

Table 3. Existing classification frameworks that correspond with the research questions

Research question	Existing classification framework to be applied	How the classification framework will be applied
What is the total number of studies published by year?	Not specified	The year that the eligible study was published will be captured.
What terms are being used to describe the nature of the research?	Not specified	The terminology used by study authors to describe the aims or methodologies used in their study will be captured e.g. infoveillance, netnography, social listening.
Which academic communities are most active in conducting health research studies that use data from social media?	Scimago Journal Subject Areas [40] including 27 subject areas including medicine, computer science, health professions, business management and accounting as well as social sciences.	The journal in which eligible study is published, along with the affiliation of all authors as well as any sources of funding for the study (if shown) will be classified according to the disciplinary taxonomy used by the Scimago Journal ranking portal. These categories are not mutually exclusive.
Where is the affiliation of the first author located?	World Bank Regional and Lending Groups for Countries includes seven regions and four lending groups [41]	The geographical location of the first author conducting the eligible study will be classified according to the regional and lending groups provided by the World Bank for 2017. These categories are not mutually exclusive.
What is the geographical scope of the sample of social media data analysed in the study?	World Bank Regional and Lending Groups for Countries includes seven regions and four lending groups [41]	The geographical location of the population sample analysed within the study will be classified according to the regional and lending groups provided by the World Bank for 2017. These categories are not mutually exclusive.
What is the purpose for which social media data are being used in the research?	Not specified	The purpose of the eligible study will be captured.
Which health topics are being studied?	The 19 health related topics which inform the World Health Organization's Sustainable Development Goals (SDG) [42]	The type of condition and health topic being studied in the eligible study will be classified according to the list of health related topics, which inform the WHO SDG. These categories are not mutually exclusive.
Which social media are used as a source of data?	Kaplan and Haenlein's eight types of social media [1]	The type of social media from which the data for the eligible study was sourced will be classified according to Kaplan and Haenlein's eight types of social media. The name of the social media will also be captured.
How large are the studies and what is the unit of analysis applied?	Not specified	The sample size and unit of analysis of the eligible study will be captured.

For peer review only

Research question	Existing classification framework to be applied	How the classification framework will be applied
How is the data from social media extracted and analysed and which proprietary tools were used?	Several types of analysis may be performed on social media data, ranging from simple descriptive statistics to qualitative research to automated real-time analytics at scale as described by Batrinca and Treleaven [43]. These types of analysis have been described in terms of computational techniques, such as natural language processing, and purposive approaches such as news analytics, opinion mining, data scraping, sentiment analysis and text analytics to name but a few.	Batrinca and Treleaven taxonomy of social media analysis techniques will be used to guide our classification of the broad approaches and specific techniques demonstrated in the eligible studies. This taxonomy may be refined as a result of new insights emerging during data extraction. We will also capture the named type of analysis performed in the eligible study as well as any reference to proprietary tools used.
How are ethical considerations applied in the published research?	Conway's taxonomy of ethics concepts for the use of Twitter in public health surveillance and research [44] and can be applied across all manner of social media.	The application of ethical concepts in the eligible studies will be classified according to the ten high level categories identified by Conway. These categories are not mutually exclusive.

Stage 5: collating, summarizing and reporting the results
The extracted data will be tabulated, with rows relating to articles, columns to classification variables and cells containing the relevant information. Both frequency analysis and trend analysis will be used to chart the classified results.

Frequency analysis
Using frequency analysis, the counts and percentages of eligible studies will be calculated. Studies will be grouped based on the classification frameworks and taxonomies applied. The result of this analysis will be a map of eligible studies represented in bubble plot, graph or tabular form.

Trend analysis
Trend analysis will be used to present the changing frequency of research over the past 20 years, based on the aforementioned classification criteria. A map showing the characteristics of included studies will be presented using both static and multi-media visualization tools, such as an animated bubble plot or graph.

Public and Patient Involvement
No patients or members of the public were involved in the protocol design or exploratory analyses, nor do we plan to include them in the conduct of the scoping review. Our results are nevertheless likely to be of interest to citizens who use social media and our decision to examine how researchers report ethical considerations reflects this concern. Dissemination will include accessible summaries and graphics, which we intend to make available to the public via social media.

Ethics and dissemination
Although some scoping reviews include a consultation phase, this one focuses on published research and online sources already in the public domain. No ethics approval is therefore required.
The completed scoping review will be submitted for publication to a peer-reviewed, interdisciplinary Open Access journal, in addition to conferences on public health and digital research. Findings will be presented using both static and multimedia visualisation tools.

Competing interests: The authors declare no competing interests.

Contributorship: JT designed the protocol and undertook the exploratory searches, with input from CP. The manuscript was drafted by JT and edited by CP.

Funding: JT is a self-funded PhD student at the University of Edinburgh, supervised by CP. She also works as a management consultant for Ernst and Young Switzerland although her employers are not involved in this research. CP is a collaborator on three RCUK-funded research programmes associated with this study: The Administrative Data Research Centre for Scotland, ESRC, Grant number ES/L007487/1; The Science and Practice of Social Machines EPSRC, EP/ J017728/1 and the Farr Institute for Health Informatics Research (Scotland) MRC, Grant number MR/K007017/1. The funders had no role in study design, data collection and analysis, decision to publish, or preparation of the manuscript.

For peer review only

Acknowledgements: Thanks to Marshall Dozier for advice on the search strategy and Santosh Vijaykumar for comments on a previous draft of the manuscript.

References

1. **Kaplan A, Haenlein M**: **Users of the world, unite! The challenges and opportunities of Social Media**. *Business Horizons* 2010, **53**(1):59-68.
2. **Complete History of Social Media: Then and Now** [https://smallbiztrends.com/2013/05/the-complete-history-of-social-media-infographic.html]
3. **Digital in 2017: Global Overview** [https://wearesocial.com/special-reports/digital-in-2017-global-overview]
4. **Social Media - Statistics and Facts** [https://www.statista.com/topics/1164/social-networks/]
5. **Current State of Social Media Research: From Practice to Theory #SMSociety15** [http://socialmedialab.ca/2015/current-state-of-social-media-research-from-practice-to-theory-part-1/]
6. **Social Media and Public Health Research** [https://bjerglund.files.wordpress.com/2012/11/final-social-media-and-public-health-research1.pdf]
7. Taylor J, Pagliari C: **Mining social media data: How are research sponsors and researchers addressing the ethical challenges?** *Research Ethics* 2017:1747016117738559.
8. Anstead N, O'Loughlin B: **Social Media Analysis and Public Opinion: The 2010 UK General Election**. *Journal of Computer-Mediated Communication* 2015, **20**(2):204-220.
9. **Social Media in Public Opinion Research: Report of the AAPOR Task Force on Emerging Technologies in Public Opinion Research** [https://www.aapor.org/AAPORKentico/AAPOR_Main/media/MainSiteFiles/AAPOR_Social_Media_Report_FNL.pdf]
10. Eysenbach G: **Infodemiology and Infoveillance: Framework for an Emerging Set of Public Health Informatics Methods to Analyze Search, Communication and Publication Behavior on the Internet**. *J Med Internet Res* 2009, **11**(1):e11.
11. Smith T: **The social media revolution**. *International Journal of Market Research* 2009, **51**(4):559.
12. Zafarani R, Ali Abbasi M, Liu H: **Social Media Mining: An Introduction**: Cambridge University Press; 2014.
13. **Social Analytics** [http://www.gartner.com/it-glossary/social-analytics]
14. Smart PR, Shadbolt NR: **Social Machines**. In: *Encyclopedia of Information Science and Technology.* edn. Edited by Khosrow-Pour M: IGI Global; 2014.
15. Kozinets RV: **Netnography**. In: *The International Encyclopedia of Digital Communication and Society.* edn.: John Wiley & Sons, Inc.; 2015.
16. Velasco E, Agheneza T, Denecke K, Kirchner G, Eckmanns TIM: **Social Media and Internet-Based Data in Global Systems for Public Health Surveillance: A Systematic Review**. *Milbank Quarterly* 2014, **92**(1):7-33.
17. Bernardo TM, Rajic A, Young I, Robiadek K, Pham MT, Funk JA: **Scoping review on search queries and social media for disease surveillance: a chronology of innovation**. *Journal of medical Internet research* 2013, **15**(7):e147.
18. Fung IC, Duke CH, Finch KC, Snook KR, Tseng PL, Hernandez AC, Gambhir M, Fu KW, Tse ZT: **Ebola virus disease and social media: A systematic review**. *American journal of infection control* 2016, **44**(12):1660-1671.
19. O'Shea J: **Digital disease detection: A systematic review of event-based internet biosurveillance systems**. *International journal of medical informatics* 2017, **101**:15-22.
20. Yan SJ, Chughtai AA, Macintyre CR: **Utility and potential of rapid epidemic intelligence from internet-based sources**. *International journal of infectious diseases : IJID : official publication of the International Society for Infectious Diseases* 2017, **63**:77-87.
21. Al-garadi MA, Khan MS, Varathan KD, Mujtaba G, Al-Kabsi AM: **Using online social networks to track a pandemic: A systematic review**. *Journal of Biomedical Informatics* 2016, **62**:1-11.
22. **A systematic literature review on event-based public health surveillance systems** [http://edoc.sub.uni-hamburg.de/haw/volltexte/2012/1614/pdf/lsab12_27.pdf]
23. Guy S, Ratzki-Leewing A, Bahati R, Gwadry-Sridhar F: **Social Media: A Systematic Review to Understand the Evidence and Application in Infodemiology**. In: *Electronic Healthcare: 2012// 2012; Berlin, Heidelberg*: Springer Berlin Heidelberg; 2012: 1-8.
24. Verhoef LM, Van de Belt TH, Engelen LJLPG, Schoonhoven L, Kool RB: **Social media and rating sites as tools to understanding quality of care: a scoping review**. *Journal of medical Internet research* 2014, **16**(2):e56.

For peer review only

25. Emmert M, Sander U, Pisch F: **Eight Questions About Physician-Rating Websites: A Systematic Review**. *J Med Internet Res* 2013, **15**(2):e24.
26. Golder S, Norman G, Loke YK: **Systematic review on the prevalence, frequency and comparative value of adverse events data in social media**. *British journal of clinical pharmacology* 2015, **80**(4):878-888.
27. Kazemi DM, Borsari B, Levine MJ, Dooley B: **Systematic review of surveillance by social media platforms for illicit drug use**. *Journal of public health (Oxford, England)* 2017:1-14.
28. Tursunbayeva A, Franco M, Pagliari C: **Use of social media for e-Government in the public health sector: A systematic review of published studies**. *Government Information Quarterly* 2017, **34**(2):270-282.
29. Patel R, Chang T, Greysen SR, Chopra V: **Social Media Use in Chronic Disease: A Systematic Review and Novel Taxonomy**. *The American Journal of Medicine*, **128**(12):1335-1350.
30. Golder S, Ahmed S, Norman G, Booth A: **Attitudes Toward the Ethics of Research Using Social Media: A Systematic Review**. *Journal of medical Internet research* 2017, **19**(6):e195.
31. Arksey H, O'Malley L: **Scoping studies: towards a methodological framework**. *International Journal of Social Research Methodology* 2005, **8**(1):19-32.
32. Levac D, Colquhoun H, O'Brien KK: **Scoping studies: advancing the methodology**. *Implementation Science : IS* 2010, **5**:69-69.
33. **Chapter 11: Scoping Reviews.** [Available from https://reviewersmanual.joannabriggs.org/]
34. Armstrong R, Hall Bj Fau - Doyle J, Doyle J Fau - Waters E, Waters E: **Cochrane Update. 'Scoping the scope' of a cochrane review**. (1741-3850 (Electronic)).
35. Horsley T, Dingwall O, Sampson M: **Checking reference lists to find additional studies for systematic reviews**. *Cochrane Database of Systematic Reviews* 2011(8).
36. **Search Engine Market Share** [https://netmarketshare.com/search-engine-market-share.aspx?options=%7B%22filter%22%3A%7B%22%24and%22%3A%5B%7B%22deviceType%22%3A%7B%22%24in%22%3A%5B%22Desktop%2Flaptop%22%5D%7D%7D%5D%7D%2C%22dateLabel%22%3A%22Trend%22%2C%22attributes%22%3A%22share%22%2C%22group%22%3A%22searchEngine%22%2C%22sort%22%3A%7B%22share%22%3A-1%7D%2C%22id%22%3A%22searchEnginesDesktop%22%2C%22dateInterval%22%3A%22Monthly%22%2C%22dateStart%22%3A%222017-03%22%2C%22dateEnd%22%3A%222018-02%22%2C%22segments%22%3A%22-1000%22%7D]
37. Lam MT, McDiarmid M: **Increasing number of databases searched in systematic reviews and meta-analyses between 1994 and 2014**. *Journal of the Medical Library Association : JMLA* 2016, **104**(4):284-289.
38. **List of social networking websites** [http://en.wikipedia.org/wiki/List_of_social_networking_websites]
39. **Top 25 Health and Medical Social Media Sites** [http://mastersinhospitaladministration.com/2011/top-25-health-and-medical-social-media-sites/]
40. **Scimago Journal & Country Rank** [http://www.scimagojr.com/journalrank.php]
41. **World Bank Country and Lending Groups** [https://datahelpdesk.worldbank.org/knowledgebase/articles/906519-world-bank-country-and-lending-groups]
42. **World Health Statistics 2016 data visualizations dashboard: Monitoring health for SDGs** [http://apps.who.int/gho/data/node.sdg.tp-1?lang=en]
43. Batrinca B, Treleaven PC: **Social media analytics: a survey of techniques, tools and platforms**. *AI & SOCIETY* 2015, **30**(1):89-116.
44. Conway M: **Ethical Issues in Using Twitter for Public Health Surveillance and Research: Developing a Taxonomy of Ethical Concepts From the Research Literature**. *J Med Internet Res* 2014, **16**(12):e290.

For peer review only

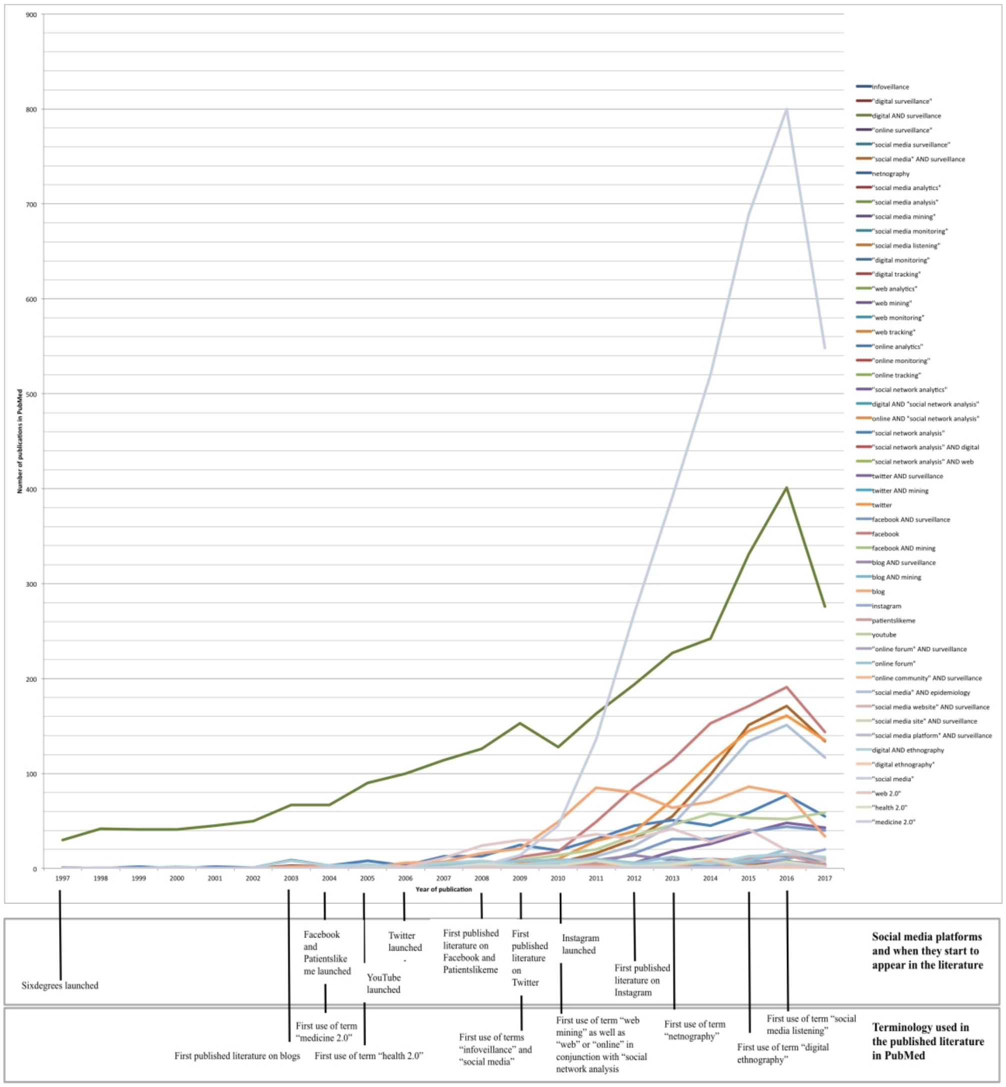

Figure 1. Results of the search queries by year

245x265mm (300 x 300 DPI)

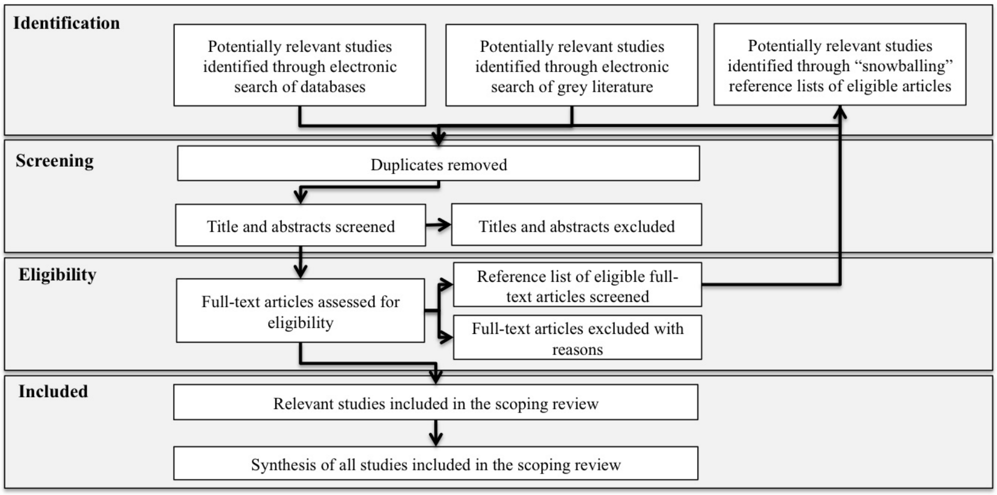

Figure 2. Steps for identification, screening, eligibility and inclusion of studies in the scoping review

113x56mm (300 x 300 DPI)

Appendix 1. Scoping and systematic reviews on the secondary use of data from social media

	Systematic or Quasi-Systematic Scoping Review	Review Aim/Objective/Main Focus	Number of Databases Used	Key Findings
1	Velasco, E., Agheneza, T., Denecke, K., Kirchner, G., Eckmanns, T.<br>Social media and internet-based data in global systems for public health surveillance: a systematic review. Milbank Q. 2014; 92(1): 7-33	To explore how useful are data from social media and the Internet, and what is the potential to enhance surveillance.	3	• No event-based surveillance systems currently used in national surveillance programs<br>• Existing systems have limitations<br>• There is a need for further development of automated technologies that monitor health-related information on the Internet<br>• The dissemination to health authorities of new information about health events is not always efficient and could be improved.<br>• No comprehensive evaluations show whether event-based surveillance systems have been integrated into actual epidemiological work during real-time health events.
2	Golder, S., Norman, G., Loke, Y.K.<br>Systematic review on the prevalence, frequency and comparative value of adverse events data in social media<br>British Journal of Clinical Pharmacology. 2015; 80(4); 878-88	To summarize the prevalence, frequency and comparative value of information on the adverse events of healthcare interventions from user comments and videos in social media.	16 databases and 2 internet search engines	• The prevalence of adverse events reports on social media varies from 0.2% to 8% of posts.<br>• 'Mild' and symptom-related adverse events are over-represented in social media and laboratory test abnormalities and 'serious' adverse events are under- represented compared with other data sources.<br>• The question as to whether searching social media for adverse events data is a valuable use of resources, resulting in improved patient outcomes remains unanswered.
3	Verhoef, L.M., Van de Belt, T.H.,<br>Engelen, L.J.L.P.G., Schoonhoven, L., Kool, R.B.<br>Social media and rating sites as tools to understanding quality of care: a scoping review<br>J Med Internet Res. 2014; 16(2); e56	To explore the relationship between data from social media and quality of care.	4	• There is a relationship between information on social media and quality of health care<br>• Drawbacks exist, since rating is anonymous and vulnerable to fraud. They are often based on only a few reviews and are predominantly positive.<br>• People providing feedback on health care via social media are presumably not always representative for the patient population.
4	Bernardo, T.M., Rajic, A., Young, I., Robiadek, K., Pham, M.T., Funk, J.A.<br>Scoping review on search queries and social media for disease surveillance: a chronology of innovation<br>J Med Internet Res. 2013; 15(7); e147	To assess the current state of knowledge regarding the use of search queries and social media for disease surveillance in order to inform future work on early detection and more effective mitigation of the effects of foodborne illness.	1 database and 2 internet search engines	• The use of search queries and social media for disease surveillance are relatively recent phenomena (first reported in 2006).<br>• Both the tools themselves and the methodologies for exploiting them are evolving over time.<br>• While their accuracy, speed, and cost compare favorably with existing surveillance systems, the primary challenge is to refine the data signal by reducing surrounding noise.
5	Fung, Tse, Cheung, Miu, & Fu. (2014). Ebola and the social media. The Lancet,	To systematically reviewed existing research pertinent to Ebola virus disease and social media, especially	6	• Included 7 articles on Twitter, 1 on Facebook, 3 on YouTube, 1 on Instagram & Flickr.

For peer review only

	Systematic or Quasi-Systematic Scoping Review	Review Aim/Objective/Main Focus	Number of Databases Used	Key Findings
	384(9961), 2207.	to identify the research questions and the methods used to collect and analyze social media.		• 11 articles examined Ebola posts' content themes, meta-data or user characteristics.<br>• One article examined how Ebola-related news videos influenced Twitter traffic.
6	Golder S, Ahmed S, Norman G, Booth A Attitudes Toward the Ethics of Research Using Social Media: A Systematic Review J Med Internet Res 2017;19(6):e195	To ascertain attitudes on the ethical considerations of using social media as a data source for research as expressed by social media users and researchers.	16 data bases and 2 internet search engines	• Attitudes varied from overly positive with people expressing the views about the essential nature of such research for the public good, to very concerned with views that social media research should not happen.<br>• Underlying reasons for this variation related to issues such as the purpose and quality of the research, the researcher affiliation, and the potential harms.<br>• The methods used to conduct the research were also important.<br>• Many respondents were positive about social media research while adding caveats such as the need for informed consent or use restricted to public platforms only.
7	Kazemi, D., Borsari, B., Levine, M., & Dooley, B. (2017). Systematic review of surveillance by social media platforms for illicit drug use. Journal of Public Health (Oxford, England), 1-14.	To review the ability of social media to better recognize illicit drug use trends was addressed.	9	Selected studies used a range of social media tools/applications, including message boards, Twitter and blog/forums/platform discussions.
8	O'Shea, Jesse. (2017). Digital disease detection: A systematic review of event-based internet biosurveillance systems. International Journal of Medical Informatics, 101, 15-22.	To update the current state of knowledge on event-based Internet biosurveillance systems by identifying all systems, including current functionality, with hopes to aid decision makers with whether to incorporate new methods into comprehensive programmes of surveillance.	3	• Each system uses different innovative technology and data sources to gather data, process, and disseminate data to detect infectious disease outbreaks.
9	Yan, Chughtai, & Macintyre. (2017). Utility and potential of rapid epidemic intelligence from internet-based sources. International Journal of Infectious Diseases, 63, 77-87.	To identify and summarise the types of Internet-based surveillance methods studied in recent literature. A secondary aim was to identify and summarise the timeliness and accuracy outcomes of Internet-based methods described in literature.	5	• Studies used search queries, social media posts and approaches derived from existing Internet-based systems for early epidemic alerts and real-time monitoring.<br>• Most studies noted improved timeliness compared to official reporting<br>• Internet-based methods showed variable correlation strength with official datasets, with some methods showing reasonable accuracy.
10	Al-Garadi, Khan, Varathan, Mujtaba, & Al-Kabsi. (2016). Using online social networks to track a pandemic: A systematic review. Journal of Biomedical Informatics, 62, 1-11.	To conduct a systematic review of the use of online social networks to track a pandemic.	5	• Identified the extent of using online social networks to track a pandemic.<br>• Online social networks contain significant information to track a pandemic almost in real time.<br>• There are challenges and future implications.
11	Agheneza T. A Systematic Review of Event-Based Public Health Surveillance Systems. Hamburg: Faculty	To identify all the event-based surveillance systems that exist and where they are based.	3	• 13 event-based systems were identified, and with 10 of the 13 systems evaluated.<br>• N. America is the leading

For peer review only

	Systematic or Quasi-Systematic Scoping Review	Review Aim/Objective/Main Focus	Number of Databases Used	Key Findings
	of Life Sciences, Hamburg University of Applied Sciences; 2011.			continent with about 77 % of the event-based systems followed by Europe and lastly Asia. No system was found in Africa, Australia and S. America.
12	Emmert M, Sander U, Pisch F. Eight questions about physician-rating websites: a systematic review. J Med Internet Res. 2013;15(2):e24.	To identify frequently discussed issues about physician-rating websites.	7	• The current usage of physician-rating websites is still low but is increasing.<br>• International data show that 1 out of 6 physicians has been rated, and approximately 90% of all ratings on physician-rating websites were positive.<br>• Could not find any evidence of "doctor-bashing".<br>• Physicians should not ignore these websites, but rather, monitor the information available and use it for internal and external purposes.
13	Guy S, Ratzki-Leewing A, Bahati R, Gwadry-Sridhar F. Social media: A systematic review to understand the evidence and application in infodemiology. Lect Notes Inst Comput Sci Soc Informatics Telecommun Eng. 2012;91:1–8.	To systematically review the literature utilizing social media as a source for disease prediction and surveillance.	2	• Open-source micro-blogging sites can be used for influenza-like-illness monitoring.
14	Tursunbayeva, Franco, & Pagliari. (2017). Use of social media for e-Government in the public health sector: A systematic review of published studies. Government Information Quarterly, 34(2), 270-282.	To investigate the adoption and use of social media by public health organizations	4	• Evidence-base is mostly descriptive, unidisciplinary and lacks the theoretical depth seen in other branches of e-Government research.<br>• The lack of robust evidence makes it difficult to draw conclusions about the effectiveness of these approaches in the public health sector
15	Patel, Chang, Greysen, & Chopra. (2015). Social Media Use in Chronic Disease: A Systematic Review and Novel Taxonomy. The American Journal of Medicine, 128(12), 1335-1350.	To evaluate clinical outcomes from applications of contemporary social media in chronic disease; to develop a conceptual taxonomy to categorize, summarize, and then analyze the current evidence base; and to suggest a framework for future studies	1	• The overall impact of social media on chronic disease was variable.<br>• Among studies that showed benefit, most used either Facebook or blogs, and were based within the domain of support.

For peer review only

Appendix 2. Total number of publications by search query

**Search query**	**Total number of publications from 1997-2017**
"social media" AND (health OR illness OR disease) AND human	3425
digital AND surveillance AND (health OR illness OR disease) AND human	2935
facebook AND (health OR illness OR disease) AND human	939
twitter AND (health OR illness OR disease) AND human	709
"social media" AND surveillance AND (health OR illness OR disease) AND human	665
blog AND (health OR illness OR disease) AND human	602
"social media" AND epidemiology AND (disease OR health OR illness) AND human	581
"social network analysis" AND (disease OR health OR illness) AND human	462
youtube AND (health OR illness OR disease) AND human	351
"web 2.0" AND (health OR illness OR disease) AND human	312
facebook AND surveillance AND (health OR illness OR disease) AND human	206
twitter AND surveillance AND (health OR illness OR disease) AND human	187
digital AND ethnography AND (health OR illness OR disease) AND human	110
"online forum" AND (health OR illness OR disease) AND human	103
blog AND surveillance AND (health OR illness OR disease) AND human	76
patientslikeme AND (health OR illness OR disease) AND human	60
"online monitoring" AND (disease OR health OR illness) AND human	48
"health 2.0" AND (health OR illness OR disease) AND human	46
twitter AND mining AND (health OR illness OR disease) AND human	43
instagram AND (health OR illness OR disease) AND human	40
online AND "social network analysis" AND (disease OR health OR illness) AND human	29
"online forum" AND surveillance AND (health OR illness OR disease) AND human	25
"online community" AND surveillance AND (health OR illness OR disease) AND human	25
infoveillance AND (disease OR health OR illness) AND human	24
"online surveillance" AND (disease OR health OR illness) AND human	23
"social network analysis" AND web AND (disease OR health OR illness) AND human	23
"digital surveillance" AND (disease OR health OR illness) AND human	16
netnography AND (disease OR health OR illness) AND human	14
"medicine 2.0" AND (health OR illness OR disease) AND human	14
"social media analysis" AND (disease OR health OR illness) AND human	12
"online tracking" AND (disease OR health OR illness) AND human	12
"web analytics" AND (disease OR health OR illness) AND human	10
"social media analytics" AND (disease OR health OR illness) AND human	9
"social media monitoring" AND (disease OR health OR illness) AND human	9
blog AND mining AND (health OR illness OR disease) AND human	9
"web mining" AND (disease OR health OR illness) AND human	8
"digital monitoring" AND (disease OR health OR illness) AND human	7
facebook AND mining AND (health OR illness OR disease) AND human	7
"social media mining" AND (disease OR health OR illness) AND human	6
"web monitoring" AND (disease OR health OR illness) AND human	6

For peer review only

Search query	Total number of publications from 1997-2017
"social media platform" AND surveillance AND (health OR illness OR disease) AND human	6
"social media surveillance" AND (disease OR health OR illness) AND human	4
"web tracking" AND (disease OR health OR illness) AND human	4
digital AND "social network analysis" AND (disease OR health OR illness) AND human	4
"social network analysis" AND digital AND (disease OR health OR illness) AND human	4
"digital ethnography" AND (health OR illness OR disease) AND human	4
"digital tracking" AND (disease OR health OR illness) AND human	3
"online analytics" AND (disease OR health OR illness) AND human	3
"social media site" AND surveillance AND (health OR illness OR disease) AND human	3
"social media listening" AND (disease OR health OR illness) AND human	2
"social network analytics" AND (disease OR health OR illness) AND human	2
"social media website" AND surveillance AND (health OR illness OR disease) AND human	2
"web surveillance" AND (disease OR health OR illness) AND human	0
"social network surveillance" AND (disease OR health OR illness) AND human	0
"social media observing" AND (disease OR health OR illness) AND human	0
"social media tracking" AND (disease OR health OR illness) AND human	0
"digital observing" AND (disease OR health OR illness) AND human	0
"digital analytics" AND (disease OR health OR illness) AND human	0
"digital mining" AND (disease OR health OR illness) AND human	0
"digital listening" AND (disease OR health OR illness) AND human	0
"web observing" AND (disease OR health OR illness) AND human	0
"web listening" AND (disease OR health OR illness) AND human	0
"online observing" AND (disease OR health OR illness) AND human	0
"online mining" AND (disease OR health OR illness) AND human	0
"online listening" AND (disease OR health OR illness) AND human	0
"social network observing" AND (disease OR health OR illness) AND human	0
"social network mining" AND (disease OR health OR illness) AND human	0
"social network monitoring" AND (disease OR health OR illness) AND human	0
"social network listening" AND (disease OR health OR illness) AND human	0
"social network tracking" AND (disease OR health OR illness) AND human	0
linkedin AND (health OR illness OR disease) AND human	0
"sixdegrees" AND (health OR illness OR disease) AND human	0

For peer review only
